# Evaluation of the Global *S*-Entropy Production in Membrane Transport of Aqueous Solutions of Hydrochloric Acid and Ammonia

**DOI:** 10.3390/e22091021

**Published:** 2020-09-12

**Authors:** Kornelia M. Batko, Andrzej Ślęzak

**Affiliations:** 1 Department of Business Informatics, University of Economics, 40287 Katowice, Poland; 2 Department of Health Science, Jan Dlugosz University, 13/15 Armia Krajowa Al., 42200 Częstochowa, Poland; aslezak52@gmail.com

**Keywords:** membrane transport, entropy production, Kedem-Katchalsky equations, concentration polarization, osmosis, diffusion, gravitational convection

## Abstract

The results of experimental studies of volume osmotic fluxes ($J_{vk}^{r}$) and fluxes of dissolved substances ($J_{k}^{r}$) in a system containing a synthetic Nephrophan^®^ membrane (Orwo VEB Filmfabrik, Wolfen, Germany) set in a horizontal plane are presented. The membrane separated water and aqueous HCl or ammonia solutions or aqueous ammonia and HCl solutions. It was found that for the homogeneity conditions of the solutions $J_{vk}$ and $J_{k}$ depend only on the concentration and composition of the solutions. For concentration polarization conditions (where concentration boundary layers are created on both sides), $J_{vk}^{r}$ and $J_{k}^{r}$ depend on both the concentration and composition of the solutions and the configuration of the membrane system. The obtained results of the $J_{vk}$ and $J_{k}$ flux studies were used to assess the global production of entropy for the conditions of homogeneity of solutions ($\Phi_{Sk}$), while $J_{vk}^{r}$ and $J_{k}^{r}$—to assess the global production of entropy for concentration polarization conditions $(\Phi_{Sk}^{r}$). In addition, the diffusion-convective effects and the convection effect in the global source of entropy were calculated. The concentration polarization coefficient $\zeta_{i}^{r}$ was related to modified concentration Rayleigh number, e.g., the parameter controlling the transition from non-convective (diffusive) to convective state. This number acts as a switch between two states of the concentration field: convective (with a higher entropy source value) and non-convective (with a lower entropy source value). The operation of this switch indicates the regulatory role of earthly gravity in relation to membrane transport.

## 1. Introduction

Membrane transport processes belong to the group of basic phenomena occurring at the level of organization of physicochemical systems, in which the membrane constitutes a selective barrier separating the interior of the system from its surroundings [1,2,3]. The driving forces of these transport phenomena are a consequence of the occurrence of various types of physical fields, such as concentration, pressure, temperature or electric potential fields, participating in shaping the field constitution of nature [4]. The flows resulting from the action of these forces, such as diffusion or osmosis, modify the physical fields, an example of which in the case of the concentration field is concentration polarization [5,6,7,8]. This modification consists in minimizing the concentration gradients, which results in minimizing, inter alia, the osmotic and diffusion fluxes of dissolved substances and the membrane potentials [8,9]. Under certain conditions depending on the composition of solutions and the orientation of the membrane with respect to the gravity vector, concentration gradients can be reconstructed by gravitational convection [8,9]. In the case of a biological cell, the membrane plays the role of a receiver and regulator of environmental signals [10].

Certain laboratory features of biological membranes are used in membrane technologies used in various fields of science, technology and medicine, as well as in various industries [11,12]. Therefore, the aim of the research is, on the one hand, to understand the mechanisms of membrane transport, and on the other, to develop membrane technologies and techniques useful in biomedicine (hemodialyzer, controlled drug release) and industrial technologies (bioreactors, biorefineries, membrane modules for food processing and water treatment) or sewage treatment) [1,11]. Most of the film-forming materials are polymers characterized by high stability and mechanical strength (e.g., polybenzimidazole, polyamide, polytriazole, cellulose acetate or cellulose triacetate) and biodegradable (poly/lactic acid, cellulose, bacterial cellulose or chitosan) [13]. They are mainly used as materials for membrane systems based on osmosis and diffusion [14,15].

Membrane transport mechanisms are based on five thermodynamic forces (four gradients: mechanical pressure, concentration, temperature, electric potential and chemical affinity) and interconnected with them, five thermodynamic fluxes (hydraulic, diffusion, thermal energy, electric charge and reactants). The cause-effect relationships of these forces and fluxes result from simple membrane processes such as osmosis or diffusion, and cross processes such as thermo-osmosis, electrodiffusion or flow potential [1,16]. Explaining the mechanisms of membrane transport is based on the methods and laws of non-equilibrium thermodynamics [17], network thermodynamics [1,18] and statistical physics [19]. Examples include the known laws of Fick, Fourier or Ohm [1] and the Kedem-Katchalsky [17], Peusner [18], Nernst-Planck [20,21,22] and Stefan-Maxwell [20] mathematical equations. In practice, it uses two groups of membrane techniques, created on the basis of the criterion of the type of driving force of the membrane process (e.g., ultrafiltration, reverse osmosis, pervaporation, dialysis, membrane distillation or electrodialysis) and the criterion of the size of the separated particles (nanofiltration, reverse osmosis and microfiltration) [12].

In thermodynamic systems, including membrane systems, internal energy can be converted into free energy and dissipated energy. The energy dissipated is the product of absolute temperature (*T*) and *S*-entropy (*S*). The rate of entropy changes of the system (dS/dt) is the sum of the rate of entropy exchanged between the system and the environment ($dS_{e}/dt$) and the rate of entropy formation inside the system ($dS_{i}/dt$) [1,15]. The rate of formation or production of entropy inside the system is determined by the expression $dS_{i}/dt=\int^{\;}\varphi_{S}dV$, where $\varphi_{S}=\left(1/T\cdot V\right)/\left(dS_{i}/dt\right)$ ≥ 0—denotes the source of entropy that is the rate of *S*-entropy formation in the volume unit (*V*) of the tested system, ($\varphi_{S}$ > 0—in an irreversible process, and $\varphi_{S}$ = 0—in a reversible process) [3]. Moreover, the source of entropy ($\varphi_{S}$) satisfies the relation $\varphi_{S}=\;\sum_{k}X_{k}J_{k}\;\;0$. This relation shows that the set of thermodynamic force ($X_{k}$) causes irreversible flows conjugated with them and opposite to them, which are measured by the $J_{k}$ fluxes, reducing the value of $X_{k}$ and leading the system to the state of thermodynamic equilibrium [1,3].

For a membrane system where a Δ*x* thick membrane separates two homogeneous electrolyte solutions of different concentrations, the entropy source of the membrane itself is $\Phi_{S}=\int_{0}^{\Delta x}\varphi_{S}dx$ [17]. If the solutions contain a solvent and *k* solutes, then the global source of entropy is described by the following equation:

(1) $$\Phi_{S}={\left(\Phi_{S}\right)}_{J_{vk}}+\;\underset{k}{\sum }{\left(\Phi_{S}\right)}_{J_{k}}+{\left(\Phi_{S}\right)}_{I}=\frac{1}{T}J_{vk}(\Delta P\pm \sum_{k}\Delta \pi_{k})+\frac{1}{T}\sum_{k}J_{k}\frac{\Delta \pi_{k}}{{\bar{C}}_{k}}+IE$$

where $\Phi_{S}$—global entropy source for the conditions of the homogeneous concentration field of solutions; ${\left(\Phi_{S}\right)}_{J_{vk}}$, ${\left(\Phi_{S}\right)}_{J_{k}}$ and ${\left(\Phi_{S}\right)}_{I}$—the *S*-entropy produced by $J_{vk}$, $J_{k}$ and *I*, respectively; $J_{vk}$ and $J_{k}$—fluxes, respectively, volume solution and *k*-th solute for the conditions of homogeneity of solutions, *I*—electric current, Δ*P* and Δ*π_k_* = *RT*Δ*C_k_*—differences of hydrostatic and osmotic pressures, respectively (*RT*—the product of the gas constant and temperature, Δ*C_k_*—difference of the concentrations of the solutions), ${\bar{C}}_{k}=\left(C_{hk}-C_{lk}\right){\left[\ln\left(C_{hk}C_{lk}{}^{-1}\right)\right]}^{-1}$—the average concentration of solutes in the membrane (M). Equation (1) is reduced to the written expression for nonelectrolyte solutions when *I* = 0 and *E* = 0 [17].

$J_{vk}$, $J_{k}$ and *I* fluxes can be described by the appropriate Kedem-Katchalsky equations for the homogeneity conditions of electrolyte solutions [17]:

(2) $$J_{vk}=L_{p}\left(\Delta P\pm \underset{k}{\sum }\varepsilon_{k}\sigma_{k}\Delta \pi_{k}\pm \beta I\right)$$

(3) $$J_{k}=\underset{k,s}{\sum }\omega_{ks}\Delta \pi_{k}+J_{vk}\left(1-\sigma_{k}\right)\;{\bar{C}}_{k}+\frac{\tau_{k}}{z_{k}F}I$$

(4) $$I=\kappa \left(\beta \Delta P+\frac{\tau_{k}}{z_{k}F}\frac{\Delta \pi_{k}}{{\bar{C}}_{k}}+\Delta E\right)$$

where *L_p_*, $\sigma_{k}$ and $\omega_{ks}$—hydraulic permeability, reflection and solute permeability coefficients, $\varepsilon_{k}$ (1 ≤ $\varepsilon_{k}\;$≤ 2)—stands for the Vant Hoff coefficient, *β*—electroosmotic coefficient, *i*—represent electric current through the membrane, $\tau_{k}$—transference number of ions, $z_{k}$—valence of ions, *F*—Faraday number, κ—conductance coefficient, Δ*E*—electromotive force difference. Equations (2)–(4) reduces to the expression for nonelectrolyte when *I* = 0. Due to the lack of accumulation or depletion of ions in the electroneutral membrane and due to the electroneutrality of the solution, it can be concluded that $J_{+}$ = $J_{-}$ = $J_{k}$ (*k* = 1 or 2).

Under real conditions, the homogeneity of the solution concentration field may be disturbed by concentration polarization. As a result, concentration boundary layers are spontaneously formed on both sides of the membrane. For the conditions of concentration polarization, and for *I* = 0 or *E* = 0, Equation (1) takes the form:

(5) $$\Phi_{S}^{r}={\left(\Phi_{S}^{r}\right)}_{J_{vk}^{r}}+\underset{k}{\sum }{\left(\Phi_{S}^{r}\right)}_{J_{k}^{r}}\;=\frac{1}{T}J_{vk}^{r}(\Delta P\pm \sum_{k}\Delta \pi_{k})+\frac{1}{T}\sum_{k}J_{k}^{r}\frac{\Delta \pi_{k}}{{\bar{C}}_{k}}$$

where $\Phi_{S}^{r}$—global entropy source for the conditions of concentration polarization, ${\left(\Phi_{S}^{r}\right)}_{J_{vk}^{r}}$ is the *S*-entropy produced by $J_{vk}^{r}$, ${\left(\Phi_{S}^{r}\right)}_{J_{k}^{r}}$ is the *S*-entropy produced by $J_{k}^{r}$, $J_{vk}^{r}$ and $J_{k}^{r}$—the volume and *k*-th solute fluxes, respectively, for the concentration polarization conditions of the solutions, *r* = A or B means the configuration of the membrane system. The Kedem-Katchalsky equations for the fluxes $J_{vk}^{r}$ and $J_{k}^{r}$ and for *I* = 0 can be written as:

(6) $$J_{vk}^{r}=\zeta_{p}^{r}L_{p}\left(\Delta P\pm \underset{k}{\sum }\zeta_{vk}^{r}\varepsilon_{k}\sigma_{k}\Delta \pi_{k}\right)$$

(7) $$J_{k}^{r}=\underset{k,s}{\sum }\zeta_{ks}^{r}\omega_{ks}\Delta \pi_{k}+J_{vk}^{r}\left(1-\zeta_{a}^{r}\sigma_{k}\right)\;{\bar{C}}_{k}$$

where $\zeta_{p}^{r}$, $\zeta_{vk}^{r}$, $\zeta_{ks}^{r}$ and $\zeta_{a}^{r}$ are the hydraulic, osmotic, diffusive and adjective concentration polarization coefficients, respectively [23]. As in the previous case, due to the lack of accumulation or depletion of ions in the electroneutral membrane and the electroneutrality of the solutions, it can be assumed that $J_{+}^{r}$ = $J_{-}^{r}$ = $J_{k}^{r}$ (*k* = 1 or 2). For this reason, in the vicinity of the electroneutral membrane, there only a phenomenon of concentration polarization of the membrane having an important influence on substances “1” and/or “2” transport through the membrane. Due to the electroneutrality of the concentrated electrolyte solutions, the electric current through the membrane (electroneutral membrane without bounded ions) during the measurement is negligible (*I* = 0) [17,24].

In [4,8] it was shown that $J_{vk}^{r}$ and $J_{k}^{r}$ depend on the transport properties of the membrane, the configuration of the membrane system as well as the physicochemical properties and composition of solutions separated by the membrane. The value of these fluxes is greater under convective than in non-convective conditions. In the case of ternary solutions (consisting of water and two dissolved substances, one of which causes an increase in density and the other a decrease in density as their concentration increases), the $J_{vk}^{r}$ and $J_{k}^{r}$ fluxes are non-linear functions of the concentration difference. Due to Equation (2), the global source of entropy for the conditions of concentration polarization ($\Phi_{S}^{r}$), is a non-linear function of $J_{vk}^{r}$ and $J_{k}^{r}$ [23,25].

The aim of the present study was to determine $J_{vk}^{r}$, $J_{k}^{r}$, $J_{vk}$ and $J_{k}$ in a single-membrane system, in which the hemodialyzer biomembrane Nephrophan^®^ (Orwo VEB Filmfabrik, Wolfen, Germany) situated in the horizontal plane separates water and a ternary solution consisting of water, ammonia and/or HCl. In order to achieve this goal, the influence of the concentration of individual components of the solutions and the configuration of the membrane system on the value of $J_{vk}^{r}$, $J_{k}^{r}$, $J_{vk}$ and $J_{k}$ fluxes under the conditions of concentration polarization, respectively, and under the conditions of homogeneity of solutions were investigated. Based on the results of the $J_{vk}^{r}$ and $J_{v}$ tests, the sources of entropy ($\Phi_{Sk}$, $\Phi_{Sk}^{r}$), the diffusion-convective effects ($\Delta \Phi_{Sk}^{r}$ = $\Phi_{Sk}-\Phi_{Sk}^{r}$) and the convective effects ($\alpha_{k}$ = $\Phi_{Sk}^{A}-\Phi_{Sk}^{B}$) in the global entropy source (*k* = 1, 2 represents the component number of the solution and *r* = A, B—configuration of the membrane system). The experiments were performed under the conditions of *E* = 0 and *I* = 0.

## 2. Model of the Electrochemical Membrane Cell

**Figure 1:**
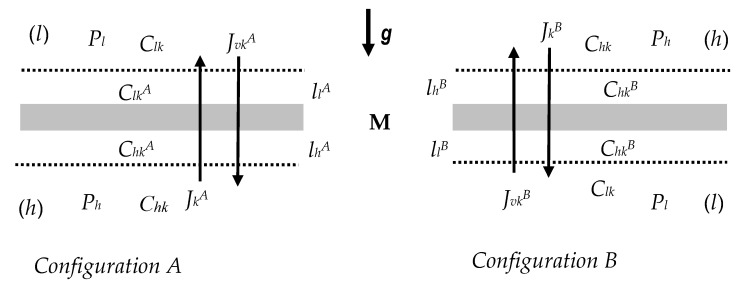
The model of single-membrane system: M—membrane, *g*—gravitational acceleration, $\;l_{l}^{A}$ and $l_{h}^{A}$ —the concentration boundary layers in configuration A, $l_{l}^{B}$ and $l_{h}^{B}$ —the concentration boundary layers in configuration B, *P_h_* and *P_l_*—mechanical pressures, *C_kh_* and *C_kl_*—global solution concentrations (*C_hk_* > *C_lk_*), $C_{lk}^{A}$, $C_{hk}^{A}$, $C_{lk}^{B}$ and $C_{hk}^{B}$ —local (at boundaries between membrane and CBLs) solution concentrations, $\;J_{vk}^{A}$ —solute and volume fluxes in configuration A, $J_{vk}^{B}$ —solute and volume fluxes in configuration B, (*k* = 1 or 2).

The subject of considerations, as well as several of our previous works, is transport in a membrane system illustrated schematically in Figure 1 [4,26]. This figure shows a model of a membrane system in which the membrane (M), situated in the horizontal plane, separates two solutions with the initial concentrations *C_hk_* and *C_lk_* (*C_hk_* > *C_lk_*, *k* = 1, 2). In configuration A, in the compartment above the membrane there is a solution with a concentration of *C_lk_*, and in the compartment under the membrane—a solution with a concentration of *C_hk_*. In configuration B—solutions with the concentration of *C_lk_*, and *C_hk_* are changed places. If we assume that the driving force for osmotic flows is the difference in concentrations between the solutions filling the upper and lower compartments, then Δ*C_k_* for configuration A has a negative sign, and for configuration B—positive.

According to the laws of diffusion, water and substances dissolved in it, penetrating through the membrane, causing the phenomenon of concentration polarization, form, on both its sides, concentration boundary layers $l_{h}^{r}$ and $l_{l}^{r}$ (*r* = A, B) with thicknesses respectively $\delta_{h}^{r}$ and $\delta_{l}^{r}$. The consequence of the formation of these layers is the reduction of the concentration difference from the value of *C_hk—_C_lk_* to the value of $C_{hk}^{r}-C_{lk}^{r}$, where $C_{hk}^{r}$ > $C_{lk}^{r}$, *C_hk_* > $C_{hk}^{r}$ and $C_{lk}^{r}$ > C_lk_.

In the case when a solution with a lower density is placed in the compartment under the membrane, and a solution with a higher density in the compartment above the membrane, the system $l_{h}^{r}$/M/$l_{l}^{r}$ loses hydrodynamic stability and, consequently, gravitational convection may occur in the concentration boundary layers region [27,28,29,30,31,32]. It appears when the thickness of the boundary concentration layers ($\delta_{h}^{r}$, $\delta_{l}^{r}$) exceeds the critical value (δ) and/or the concentration polarization coefficients ($\zeta_{k}^{r}$) exceeds the critical value ($\zeta_{k}$) and when the concentration Rayleigh number (*R_Ck_*) that control the process of the appearance of gravitational convection, will exceed their critical values [28,33,34]. The concentration Rayleigh number for membrane transport processes of ternary solutions can be represented by the expressions [35,36]:

(8) $$R_{C1}=\frac{gD_{1}{}^{2}}{16{\left(RT\right)}^{3}\rho_{0}\nu_{0}\omega_{1}{}^{3}}\left[\frac{\partial \rho }{\partial C_{1}}\;\left(1-\zeta_{1}\right)\left(C_{h1}-C_{l1}\right)+\frac{\partial \rho }{\partial C_{2}}\;\left(1-\zeta_{2}\right)\left(C_{h2}-C_{l2}\right)\right]{\left(\frac{1-\zeta_{1}}{\zeta_{1}}\right)}^{3}$$

where $R_{C1}$—concentration Rayleigh Number, $\rho_{0}$—mass density, $\nu_{0}$—kinematic viscosity of solution, *RT*—product of the gas constant and temperature, $\omega_{1}$—solute permeability coefficient, g—gravitational acceleration, $\partial \rho /\partial C_{k}$—variation of density with concentration, $\zeta_{1}$—concentration polarization coefficient, $D_{1}$—diffusion coefficient, (*k* = 1, 2). It is worth noting that Equation (8) does not contain the concentration thickness of the boundary layer (δ). To get $R_{C2}$ it is enough to change the index “1” to “2”.

Over time, the destructive effect of gravitational convection limits the growth of $\delta_{h}^{r}$ and $\delta_{l}^{r}$ and accelerates the diffusion of substances beyond the layers, which extends the effect of convection to the entire volume of the solution. Under certain conditions, even liquid structuring may occur, which is manifested in the appearance of “plum structures” [37,38].

The process of creating concentration boundary layers is accompanied by a decrease in the volume osmotic fluxes from $J_{vk}$ to $J_{vk}^{r}$ and the solute fluxes from $J_{k}$ to $J_{k}^{r}$ [7]. Using Equations (1) and (5), the global source of entropy for ternary solutions can be represented as:

(9) $$\Phi_{Sk}^{r}={\left(\Phi_{S}^{r}\right)}_{J_{vk}^{r}}+\overset{2}{\underset{k=1}{\sum }}{\left(\Phi_{S}^{r}\right)}_{J_{k}^{r}}=\frac{1}{T}J_{vk}^{r}\left[\Delta P\pm RT\overset{2}{\underset{k=1}{\sum }}\left(C_{hk}-C_{lk}\right)\right]+R\overset{2}{\underset{k=1}{\sum }}J_{k}^{r}\;ln\frac{C_{hk}}{C_{lk}}\;\;$$

(10) $$\Phi_{Sk}={\left(\Phi_{S}\right)}_{J_{vk}}+\overset{2}{\underset{k=1}{\sum }}{\left(\Phi_{S}\right)}_{J_{k}}=\frac{1}{T}J_{vk}\left[\Delta P\pm RT\overset{2}{\underset{k=1}{\sum }}\left(C_{hk}-C_{lk}\right)\right]+R\overset{2}{\underset{k=1}{\sum }}J_{k}\;ln\frac{C_{hk}}{C_{lk}}\;\;$$

To calculate the sources of entropy $\Phi_{Sk}$ and $\Phi_{Sk}^{r}$, it is enough to experimentally determine the concentration dependences of the fluxes $J_{vk}^{r}$, $J_{vk}$, $J_{k}^{r}$ and $J_{k}$.

## 3. Methodology for Measuring the Volume Osmotic and Solute Fluxes

The study of volume osmotic transport and transport of dissolved substances was carried out using the measuring set described in a previous paper [29]. The set consisted of two cylindrical measuring vessels with a volume of 200 cm^3^ each. One of the vessels contained the tested binary solution (aqueous HCl or NH_3_·H_2_O solution) or ternary (aqueous solution of HCl and NH_3_·H_2_O). In turn, the second vessel in all experiments contained an aqueous solution of HCl and/or NH_3_·H_2_O (NH_4_OH) with a constant concentration *C_l_*_1_ = *C_l_*_2_ = 1 mol m^−3^. The solutions in the vessels were separated by the Nephrophan^®^ (Orwo VEB Filmfabrik, Wolfen, Germany) biomembrane, set in a horizontal plane, with an area of *A* = 3.36 cm^2^ and transport properties determined by the following factors: hydraulic permeability (*L_p_*), reflection (*σ*) and diffusion permeability (*ω*). The values of these coefficients for HCl (index 1) and NH_3_·H_2_O (index 2), determined in a series of independent experiments carried out according to the procedure described in paper [16], were: *L_p_* = 5 × 10^−12^ m^3^N^−1^s^−1^, *σ*_1_ = 0.06, *σ*_2_ = 0.01, *ω*_11_ = 1.24 × 10^−9^ mol N^−1^s^−1^, *ω*_12_ = 1.4 × 10^−12^ mol N^−1^s^−1^, *ω*_22_ = 2.68 × 10^−9^ mol N^−1^s^−1^ and *ω*_21_ = 2.5 × 10^−12^ mol N^−1^s^−1^. Nephrophan^®^ (Orwo VEB Filmfabrik, Wolfen, Germany) is a microporous, highly hydrophilic and electroneutral membrane made of regenerated cellulose [39].

A graduated (every 0.5 mm^3^) pipette set in a plane parallel to the plane of the membrane was connected to the vessel containing the higher concentration. The change in volume (Δ*V^r^*) of the solution in this vessel of the plumbing system was measured with this pipette. In turn, the second vessel was connected to a reservoir of an aqueous solution of HCl and/or NH_4_OH (NH_3_·H_2_O) with a concentration of *C_l_*_1_ = *C_l_*_2_ = 1 mol m^−3^, with adjustable height relative to the pipette. This made it possible to compensate for the hydrostatic pressure (Δ*P* = 0) present in the measurement set. The measurements were performed according to the procedure described in [8], which consisted of two stages. In the first stage, the increases of Δ*V^r^* were measured under the conditions of intensive mechanical stirring of the solutions with an angular speed of 500 rpm. The second stage started as soon as steady-state flows were achieved, and the stirring of the solutions was turned off. In this step, the increases of Δ*V^r^* were also measured until the steady state of the flows was obtained. Each experiment was performed for configurations A and B of the membrane system. In configuration A, the test solution was filled into the vessel under the membrane, and in configuration B—the vessel over the membrane. It should be noted that the volume flows took place from the vessel with a lower concentration of solutions to the vessel with a higher concentration of solutions, and the flows of dissolved substances in the opposite direction. Therefore, it was assumed that in the configuration A the fluxes $J_{vk}^{r}$, $J_{vk}$, $J_{k}^{r}$ and $J_{k}$ and the concentration differences Δ*C_k_* (*k* = 1, 2) are negative ($J_{vk}^{r}$, $J_{vk}$—directed vertically downwards, $J_{k}^{r}$ and $J_{k}$—vertically upwards), and in configuration B—positive ($J_{vk}^{r}$, $J_{vk}$—vertically upwards, $J_{k}^{r}$, $J_{k}$—vertically downwards).

The tests were carried out in isobaric-isothermal conditions for *T* = 295 K and Δ*P* = 0. The volume flow was calculated on the basis of the volume changes (Δ*V^r^*) in the pipette over time ∆t through the membrane surface *S*, using the formula $J_{vk}^{r}$ = ($\Delta V^{r}$)*S*^−1^(Δ*t*)^−1^ (*r* = A, B). Flows of dissolved substances were calculated on the basis of the formula $J_{k}^{r}$ = $\left(\Delta C_{k}^{r}V_{u}\right)$*S*^−1^(Δ*t*)^−1^ (*k* = 1, 2; *r* = A, B),$\;V_{u}$—volume of the measuring vessel, $\Delta C_{k}^{r}$—global concentration exchanes in the solutions studied was performer by the standard physico-chemical method [40,41]. In this expression, due to the lack of accumulation or depletion of ions inside the electroneutral membrane and in its surroundings (electroneutral solutions), we assume that $J_{+}^{r}$ = $J_{-}^{r}$ = $J_{1}^{r}$ $(J_{1}^{r}\equiv J_{HCl}^{r}$), $J_{+}^{r}$ = $J_{-}^{r}$ = $J_{2}^{r}$ $(J_{2}^{r}\equiv J_{NH_{4}OH}^{r}$), $\Delta C_{+}^{r}$ = $\Delta C_{-}^{r}$ = $\Delta C_{1}^{r}$ ($\Delta C_{1}^{r}\equiv \;\Delta C_{HCl}^{r}$) and $\Delta C_{+}^{r}$ = $\Delta C_{-}^{r}$ = $\Delta C_{2}^{r}$ ($\Delta C_{2}^{r}\equiv \;\Delta C_{NH_{4}OH}^{r}$).

The study of volume flows and flows of dissolved substances in both configurations consisted in determining the characteristics $J_{vk}$ = *f*(*t*), $J_{vk}^{r}$ = *f*(*t*), $J_{k}$ = *f*(*t*) and $J_{k}^{r}$ = *f*(*t*),

(*k* = 1, 2; *r* = A, B) for different concentrations of solutions. Each measurement series was repeated 3 times. The relative error in determining $J_{vk}$, $J_{v}^{r}$, $J_{k}$ and $J_{k}^{r}$ was not greater than 5%. Based on the characteristics $J_{vk}$ = *f*(*t*), $J_{vk}^{r}$ = *f*(*t*), $J_{k}$ = *f*(*t*) and $J_{k}^{r}$ = *f*(*t*) for the steady state, the characteristics $J_{v1}=f(\Delta C_{1}$, $\Delta C_{2}$ = constant), $J_{v2}=f(\Delta C_{2}$, $\Delta C_{1}$ = constant) $J_{v1}^{r}=f(\Delta C_{1}$, $\Delta C_{2}$ = constant), $J_{v2}^{r}=f(\Delta C_{2}$, $\Delta C_{1}$ = constant), $J_{1}=f(\Delta C_{1},\;\Delta C_{2}$ = constant), $J_{2}=f(\Delta C_{2},\;\Delta C_{1}$= constant), $J_{1}^{r}=f(\Delta C_{1},\;\Delta C_{2}$ = constant) and $J_{2}^{r}=f\Delta C_{2},\;\Delta C_{1}\;$= constant). Based on these characteristics, the concentration source of entropy was calculated: $\Phi_{S1}=f(\Delta C_{1},\;\Delta C_{2}$ = constant), $\Phi_{S2}=f(\Delta C_{2},\;\Delta C_{1}$ = constant), $\Phi_{S1}^{r}=f(\Delta C_{1},\;\Delta C_{2}$ = constant), $\Phi_{S2}^{r}=f(\Delta C_{2},\;\Delta C_{1}$ = constant), $\Delta \Phi_{S1}^{r}=f(\Delta C_{1},\;\Delta C_{2}$ = constant) and $\Delta \Phi_{S2}^{r}=f(\Delta C_{2},\;\Delta C_{1}$ = constant).

## 4. Results and Discussion

### 4.1. Concentration Dependencies of the Volume Osmotic Flux

**Figure 2:**
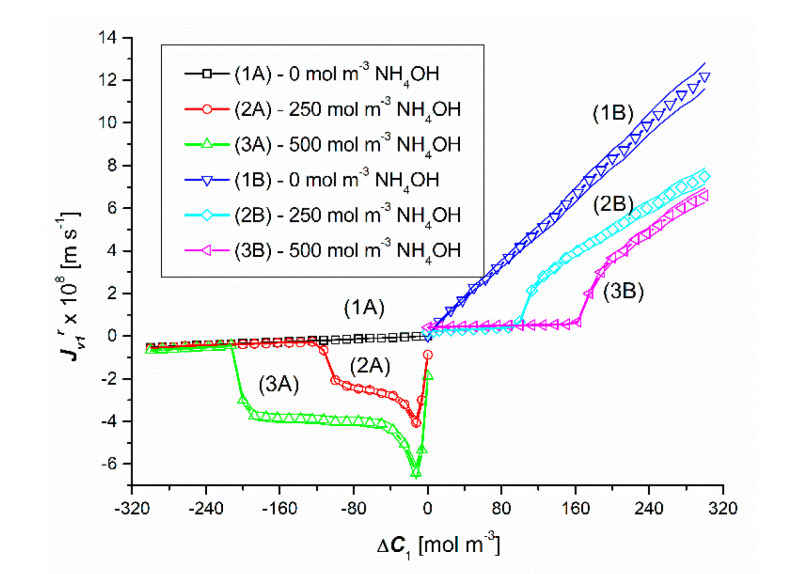
Graphical illustration of the experimental dependence $J_{v1}^{r}=f(\Delta C_{1},\;\Delta C_{2}$ = constant), for HCl solutions in NH_4_OH aqueous solution and concentration polarization conditions. Graphs 1A and 1B were obtained for Δ*C*_2_ = 0, graphs 2A and 2B—for Δ*C*_2_ = 250 mol m^−3^ and graphs 3A and 3B—for Δ*C*_2_ = 500 mol m^−3^.

**Figure 3:**
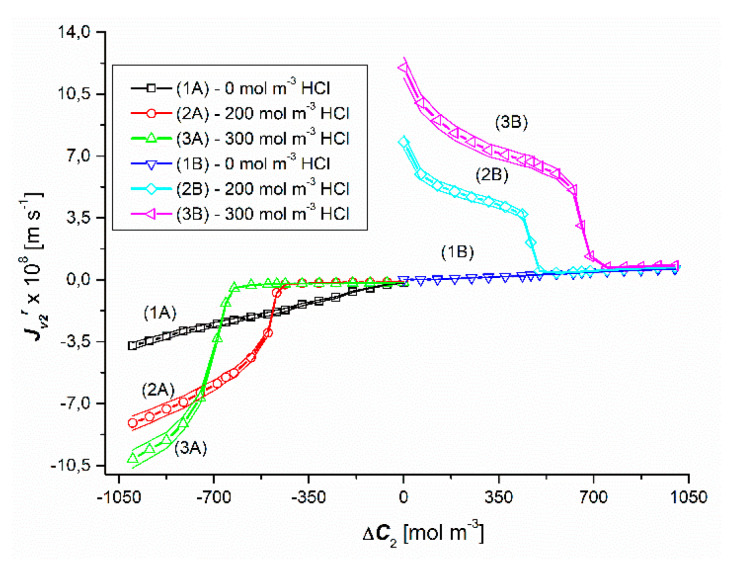
Graphical illustration of the experimental dependence $J_{v2}^{r}=f(\Delta C_{2},\;\Delta C_{1}$ = constant), for NH_4_OH solutions in an aqueous HCl solution and concentration polarization conditions. Graphs 1A and 1B were obtained for Δ*C*_1_ = 0, graphs 2A and 2B—for Δ*C*_1_ = 200 mol m^−3^ and graphs 3A and 3B—for Δ*C*_1_ = 300 mol m^−3^.

The results of the volume osmotic flux tests for the concentration polarization conditions of the solutions separated by the membrane are shown in Figure 2 and Figure 3. Figure 2 shows the experimental dependencies $J_{v1}^{r}=f(\Delta C_{1},\;\Delta C_{2}$ = constant), and in Figure 3—the experimental dependencies $J_{v2}^{r}=f(\Delta C_{2},\;\Delta C_{1}$ = constant). The graphs in the third quadrant of the coordinate system (1A, 2A, 3A) refer to configuration A of the membrane system). On the other hand, the graphs in the first quadrant of the coordinate system (1B, 2B, 3B) refer to configuration B of the membrane system.

Lines 1A and 1B shown in Figure 2 show that in the case of Δ*C*_1_ < 0 and Δ*C*_1_ > 0 (for Δ*C*_2_ = 0) in binary solutions it causes a linear decrease (graph 1A) or a linear increase (graph 1B) of the $J_{v1}^{r}$ flux value, but the slope of line 1A is less than line 1B. This is because for Δ*C*_1_ < 0 the concentration polarization minimizes Δ*C*_1_ and consequently also $J_{v1}^{r}$. On the other hand, for Δ*C*_1_ > 0, gravitational convection partially restores Δ*C*_1_, which in turn gives higher values of $J_{v1}^{r}$. On the other hand, graphs 1A and 1B presented in Figure 3 show that in the case of Δ*C*_2_ < 0 and Δ*C*_2_ > 0 (for Δ*C*_1_ = 0) in binary solutions it causes a linear increase (diagram 1A) or a linear decrease (diagram 1B) of $J_{v2}^{r}$ fluxes, but this time the slope of line 1A is greater than line 1B. In this case, for Δ*C*_2_ > 0, the concentration polarization minimizes Δ*C*_2_ and consequently also $J_{v2}^{r}$. On the other hand, for Δ*C*_2_ < 0, gravitational convection partially restores Δ*C*_2_, which in turn gives higher values of $J_{v2}^{r}$.

Lines 2A and 3A as well as 2B and 3B shown in Figure 2 show that in the case of Δ*C*_1_ < 0 and Δ*C*_1_ > 0 (for Δ*C*_2_ = 250 mol m^−3^ and Δ*C*_2_ = 500 mol m^−3^) in ternary solutions, changes in Δ*C*_1_ cause various changes of $J_{v1}^{r}$ fluxes. These graphs show that for Δ*C*_1_ < 0 an initial decrease and then a non-linear increase in the value of $J_{v1}^{r}$ fluxes are observed. Graphs 2B and 3B show that for Δ*C*_1_ > 0, after the initial small linear, there is a non-linear increase in the value of the flux $J_{v1}^{r}$. Moreover, comparing the graphs 1A, 2A and 3A for the test results presented in this figure, the following relationships are satisfied: $J_{v1}^{r}$ (for Δ*C*_2_ = 0) > $J_{v1}^{r}$ (for Δ*C*_2_ = 250 mol m^−3^) > $J_{v1}^{r}$ (for Δ*C*_2_ = 500 mol m^−3^). On the other hand, the comparison of the graphs 1B, 2B and 3B shows that $J_{v1}^{r}$ (for Δ*C*_2_ = 0) > $J_{v1}^{r}$ (for Δ*C*_2_ = 250 mol m^−3^) > $J_{v1}^{r}$ (for Δ*C*_2_ = 500 mol m^−3^). The jump in the value of $J_{v1}^{r}$ is caused by the transition of the system from non-convective to convective state. In turn, the abrupt decrease in the value of $J_{v1}^{r}$ is caused by the transition of the system from convective to non-convective state.

Comparing the curves 2A and 2B as well as 3A and 3B shown in Figure 2, it can be seen that in the case of the first pair of curves, for Δ*C*_1_ = ±107.7 mol m^−3^, the $J_{v1}^{r}$ fluxes are equal in value ($J_{v1}^{r}$ = ±1.29 × 10^−8^ m s^−1^), but directed in the opposite direction. On the other hand, in the case of the second pair of curves, the $J_{v1}^{r}$ fluxes are equal in value ($J_{v1}^{r}$ = ±1.46 × 10^−8^ m s^−1^) and directed opposite for Δ*C*_1_ = ±206.2 mol m^−^^3^. The equality of the volume osmotic flux means that the volume osmotic flux is independent of the configuration of the membrane system. This means that the diaphragm system does not discriminate in the gravity direction. Moreover, for Δ*C*_1_ > −107.7 mol m^−3^ and Δ*C*_1_ > −206.2 mol m^−3^, membrane transport in configuration A of the membrane system and for Δ*C*_1_ > 107.7 mol m^−3^ and Δ*C*_1_ > 206.2 mol m^−3^ (for configuration B) takes place under the conditions of concentration polarization destruction by free convection and is osmotic-diffusion-convective in nature. In turn, for Δ*C*_1_ < −107.7 mol m^−3^ and Δ*C*_1_ < −206.2 mol m^−3^ in the membrane transport (in configuration A) of the membrane system and for Δ*C*_1_ > 107.7 mol m^−3^ and Δ*C*_1_ < 206.2 mol m^−3^ (in configuration A) B of the membrane system) takes place under the conditions of concentration polarization and is osmotic and diffusive.

Plots 2A and 3A as well as 2B and 3B shown in Figure 3 show that in the case of Δ*C*_2_ < 0 and Δ*C*_2_ > 0 (for Δ*C*_1_ = 200 mol m^−3^ and Δ*C*_1_ = 300 mol m^−3^) in ternary solutions, changes in Δ*C*_2_ cause different changes of $J_{v2}^{r}$ fluxes. These graphs show that for Δ*C*_2_ < 0, an initial slight linear and then a non-linear decrease in the values of $J_{v2}^{r}$ fluxes is observed. Graphs 2B and 3B show that for Δ*C*_2_ > 0, with an increase in the value of Δ*C*_2_, there is a non-linear decrease in the value of the flux $J_{v2}^{r}$. Moreover, comparing the graphs 1A, 2A and 3A for the test results presented in this figure, the relations between $J_{v2}^{r}$ (for Δ*C*_1_ = 0), $J_{v2}^{r}$ (for Δ*C*_1_ = 200 mol m^−3^) and $J_{v2}^{r}$ (for Δ*C*_1_ = 300 mol m^−3^) are different depending on the Δ*C*_2_ range. On the other hand, the comparison of graphs 1B, 2B and 3B shows that $J_{v2}^{r}$ (for Δ*C*_1_ = 0) < $J_{v2}^{r}$ (for Δ*C*_1_ = 200 mol m^−3^) < $J_{v2}^{r}$ (for Δ*C*_1_ = 300 mol m^−3^). As in the previous case, the jump or decrease in the value of $J_{v2}^{r}$ is caused by the transition of the system from non-convective to convective state or the other way.

Comparing the curves 2A and 2B as well as 3A and 3B presented in Figure 3, it can be seen that in the case of the first pair of curves, for Δ*C*_2_ = ±476.7 mol m^−3^, the $J_{v2}^{r}$ fluxes are equal in value ($J_{v2}^{r}$ = ±1.5 × 10^−8^ m s^−1^), but directed in the opposite direction. On the other hand, in the case of the second pair of curves, the $J_{v2}^{r}$ fluxes are equal in value ($J_{v2}^{r}$ = ±2.19 × 10^−8^ m s^−1^) and directed opposite for Δ*C*_2_ = ±664 mol m^−3^. The equality of the volume osmotic fluxes means that the volume osmotic flux is independent of the configuration of the membrane system and thus of the gravity direction. Moreover, for Δ*C*_2_ > −476.7 mol m^−3^ and Δ*C*_2_ > −664 mol m^−3^, membrane transport in configuration A of the membrane system and for Δ*C*_2_ < 476.7 mol m^−3^ and Δ*C*_2_ < 664 mol m^−3^ (for configuration B) takes place under the conditions of concentration polarization destruction by gravitational convection and is osmotic-diffusion-convective in nature. In turn, for Δ*C*_2_ > −476.7 mol m^−3^ and Δ*C*_2_ > −664 mol m^−3^ in the membrane transport (in configuration A) of the membrane system and for Δ*C*_2_ < 476.7 mol m^−3^ and Δ*C*_2_ < 664 mol m^−3^ (in B of the membrane system) takes place under the conditions of concentration polarization and is osmotic and diffusive.

**Figure 4:**
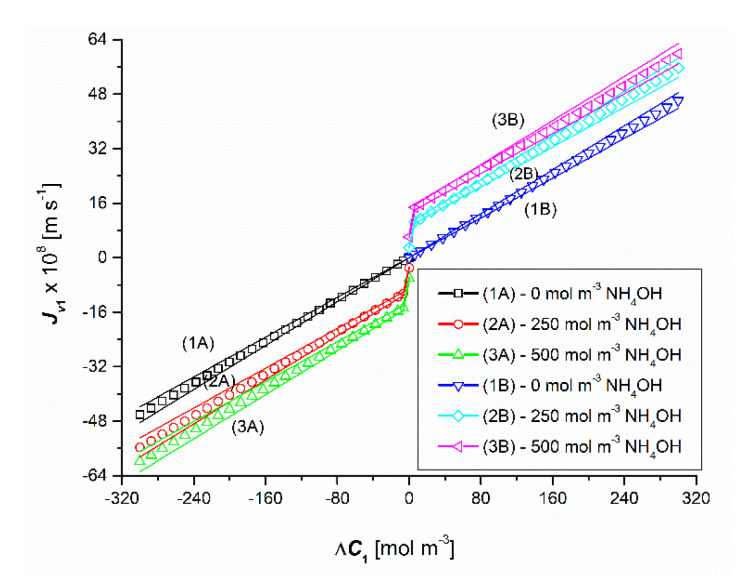
Graphical illustration of the experimental dependence $J_{v1}=f(\Delta C_{1},\;\Delta C_{2}$ = constant), for HCl solutions in NH_4_OH aqueous solution and the conditions of homogeneity of the solutions. Graphs 1A and 1B were obtained for Δ*C*_2_ = 0, graphs 2A and 2B—for Δ*C*_2_ = 250 mol m^−3^ and graphs 3A and 3B—for Δ*C*_2_ = 500 mol m^−3^.

**Figure 5:**
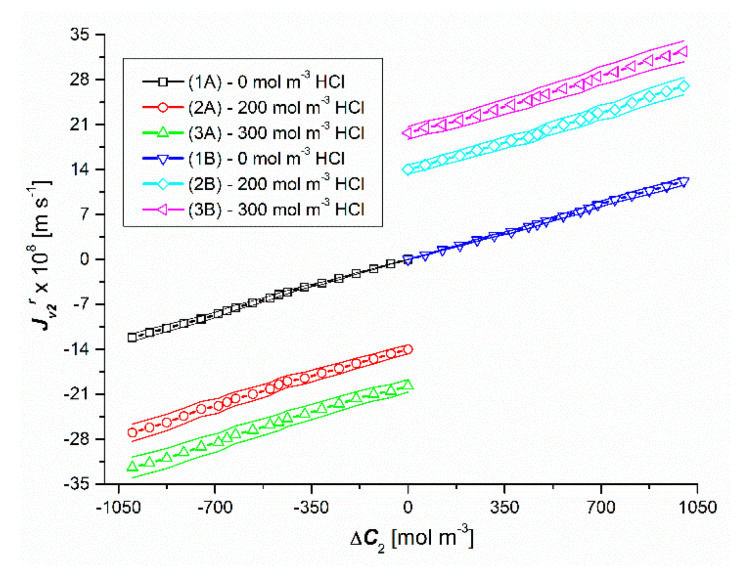
Graphical illustration of the experimental dependence $J_{v2}=f(\Delta C_{2},\;\Delta C_{1}$ = constant), for NH_4_OH solutions in an aqueous HCl solution and homogeneity conditions of the solutions. Graphs 1A and 1B were obtained for Δ*C*_1_ = 0, graphs 2A and 2B—for Δ*C*_1_ = 200 mol m^−3^ and graphs 3A and 3B—for Δ*C*_1_ = 300 mol m^−3^.

Figure 4 and Figure 5 show the results of the volume osmotic flux tests for the uniformity conditions of the solutions. Figure 4 and Figure 5 show that changing the sign of Δ*C*_1_ and/or Δ*C*_2_ changes the sign of $J_{v1}$ and $J_{v2}$ but does not change the value. This means that $J_{v1}$ and $J_{v2}$ do not depend on the configuration of the diaphragm system. Moreover,$\;J_{v1}$ is a linear (except for the initial section of plots 2 and 3) a function of Δ*C*_1_, with a fixed value of Δ*C*_2_. It should be noted that for the test results presented in Figure 4, non-zero values of Δ*C*_2_, and for the test results presented in Figure 5, non-zero values of Δ*C*_1_, cause a parallel shift of graphs 2 and 3 in relation to graph 1, while 1 is greater than plot 3 relative to 2. This is due to a 2-fold increase in the osmotic pressure difference Δ*π*_2_ due to complete dissociation of NH_3_·H_2_O (NH_4_OH) in the presence of HCl.

### 4.2. Concentration Dependencies of Solute Fluxes

**Figure 6:**
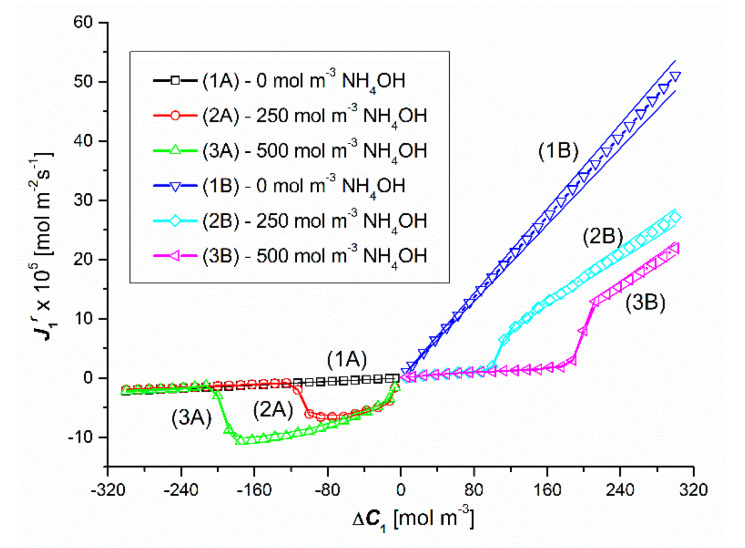
Graphic illustration of the experimental dependence $J_{1}^{r}=f(\Delta C_{1},\;\Delta C_{2}$ = constant), for HCl solutions in NH_4_OH aqueous solution and concentration polarization conditions. Graphs 1A and 1B were obtained for Δ*C*_2_ = 0, graphs 2A and 2B—for Δ*C*_2_ = 250 mol m^−3^ and graphs 3A and 3B—for Δ*C*_2_ = 500 mol m^−3^.

**Figure 7:**
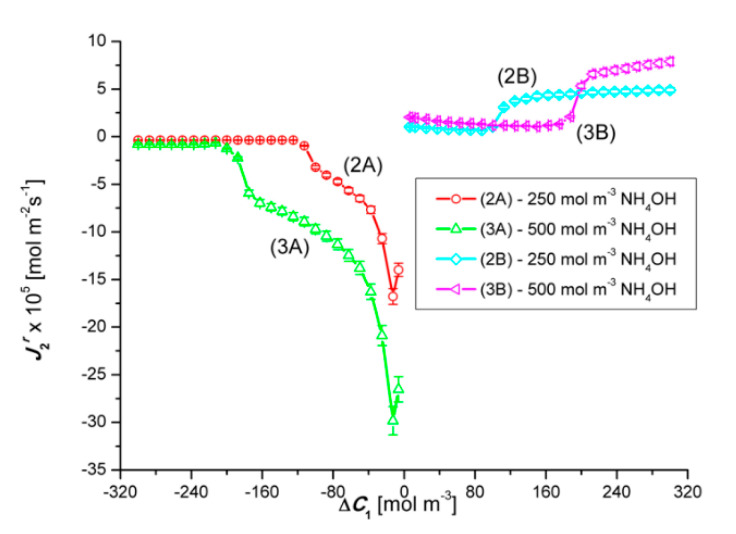
Graphical illustration of the experimental dependence $J_{2}^{r}=f(\Delta C_{1},\;\Delta C_{2}$ = constant), for NH_4_OH solutions in aqueous HCl solutions and concentration polarization conditions. Graphs 2A and 2B—for Δ*C*_2_ = 250 mol m^−3^ and graphs 3A and 3B—for Δ*C*_2_ = 500 mol m^−3^.

The results of the study of the flux of dissolved substances for the conditions of concentration polarization of the solutions separated by the membrane are shown in Figure 6and Figure 7. Figure 6 shows the experimental dependences $J_{1}^{r}=f(\Delta C_{1},\;\Delta C_{2}$ = constant), and in Figure 7, the experimental dependencies $J_{2}^{r}=f(\Delta C_{2},\;\Delta C_{1}$ = constant). The graphs in the third quadrant of the coordinate system (1A, 2A, 3A) refer to configuration A of the membrane system. In turn, the graphs in the first quadrant of the coordinate system (1B, 2B, 3B) refer to the configuration B of the membrane system. Graphs 1A and 1B shown in Figure 6 show that in the case of Δ*C*_1_ < 0 and Δ*C*_1_ > 0 (for Δ*C*_2_ = 0) in binary solutions it causes a linear decrease (graph 1A) or a linear increase (graph 1B) of the $J_{1}^{r}$. flux value, but the slope of line 1A is less than line 1B. This is because for Δ*C*_1_ < 0 the concentration polarization minimizes Δ*C*_1_ and consequently also $J_{1}^{r}$. On the other hand, for Δ*C*_1_ > 0, gravitational convection partially restores Δ*C*_1_, which consequently gives higher values of $J_{1}^{r}$. On the other hand, graphs 1A and 1B presented in Figure 7 show that in the case of Δ*C*_2_ < 0 and Δ*C*_2_ > 0 (for Δ*C*_1_ = 0) in binary solutions it causes a linear increase (graph 1A) or a linear decrease (graph 1B) of $J_{2}^{r}$ fluxes, but this time the slope of line 1A is greater than line 1B. In this case, for Δ*C*_2_ > 0, the concentration polarization minimizes Δ*C*_2_ and consequently also $J_{2}^{r}$. On the other hand, for Δ*C*_2_ < 0, gravitational convection partially restores Δ*C*_2_, which consequently gives higher values of $J_{2}^{r}$.

Graphs 2A and 3A as well as 2B and 3B shown in Figure 6 show that in the case of Δ*C*_1_ < 0 and Δ*C*_1_ > 0 (for Δ*C*_2_ = 250 mol m^−3^ and Δ*C*_2_ = 500 mol m^−3^) in ternary solutions, changes in Δ*C*_1_ cause different changes of $J_{1}^{r}$ fluxes. These graphs show that for Δ*C*_1_ < 0 an initial decrease and then a non-linear increase in the values of $J_{1}^{r}$ fluxes are observed. Graphs 2B and 3B show that for Δ*C*_1_ > 0, after the initial small linear, there is a non-linear increase in the value of the flux $J_{1}^{r}$. Moreover, comparing the graphs 1A, 2A and 3A for the test results presented in this figure, the following dependences are fulfilled: $J_{1}^{r}$ (for Δ*C*_2_ = 0) > $J_{1}^{r}$ (for Δ*C*_2_ = 250 mol m^−3^) > $J_{1}^{r}$ (for Δ*C*_2_ = 500 mol m^−3^). On the other hand, the comparison of the graphs 1B, 2B and 3B allows to conclude that $J_{1}^{r}$ (for Δ*C*_2_ = 0) > $J_{1}^{r}$ (for Δ*C*_2_ = 250 mol m^−3^) > $J_{1}^{r}$ (Δ*C*_2_ = 500 mol m^−3^). The jump in the value of $J_{1}^{r}$ is caused by the transition of the system from non-convective to convective states. In turn, the abrupt decrease in the value of $J_{1}^{r}$ is caused by the transition of the system from convective to non-convective state.

Comparing the curves 2A and 2B as well as 3A and 3B presented in Figure 6, it can be seen that in the case of the first pair of curves, for Δ*C*_1_ = ±106.7 mol m^−3^, the fluxes $J_{1}^{r}$ are equal in terms of value ($J_{1}^{r}$ = ±4.1 × 10^−5^ mol m^−2^s^−1^), but in the opposite direction. In turn, in the case of the second pair of curves, the fluxes $J_{1}^{r}$ are equal in value ($J_{1}^{r}$ = ±5.6 × 10^−5^ mol m^−2^s^−1^) and directed opposite for Δ*C*_1_ = ±194.4 mol m^−3^. The equality of the volume osmotic flux means that the volume osmotic flux is independent of the configuration of the membrane system. Moreover, for Δ*C*_1_ > −106.7 mol m^−3^ and Δ*C*_1_ > −194.4 mol m^−3^, the membrane transport in configuration A of the membrane system and for Δ*C*_1_ > 106.7 mol m^−3^ and Δ*C*_1_ > 194.4 mol m^−3^ (for configuration B) takes place under the conditions of concentration polarization destruction by free convection and is diffusive-convective in nature. In turn, for Δ*C*_1_ < −106.7 mol m^−3^ and Δ*C*_1_ < −194.4 mol m^−3^ in the membrane transport (in configuration A) of the membrane system and for Δ*C*_1_ > 106.7 mol m^−3^ and Δ*C*_1_ < 194.4 mol m^−3^ (in configuration A) B of the membrane system) takes place in the conditions of concentration polarization and is diffusive.

Figure 7 shows the results of the $J_{2}^{r}$ flux generated by the constant difference in concentrations $\Delta C_{2}$ = 250 mol m^−3^ (graphs 2A and 2B) and $\Delta C_{2}$ = 500 mol m^−3^ (graphs 3A and 3B) for a variable value of $\Delta C_{1}$. Hence, $J_{2}^{r}$ should be constant. However, adding HCl to aqueous solutions of ammonia causes an increase in the density of the solution, which in turn induces convective movements causing partial destruction of CBLs and leads to an increase in the value of $J_{2}^{r}$, depending on $\Delta C_{1}$.

**Figure 8:**
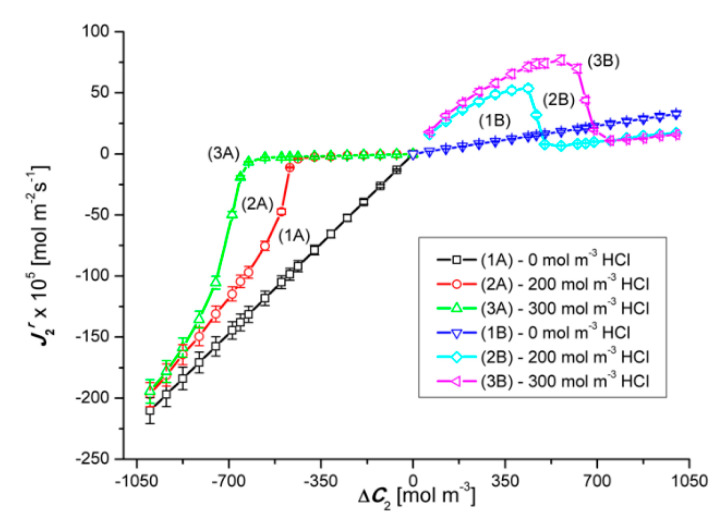
Graphical illustration of the experimental dependence $J_{2}^{r}=f(\Delta C_{2},\;\Delta C_{1}$ = constant), for NH_4_OH solutions in an aqueous HCl solution and concentration polarization conditions. Graphs 1A and 1B were obtained for Δ*C*_1_ = 0, graphs 2A and 2B—for Δ*C*_1_ = 200 mol m^−3^ and graphs 3A and 3B—for Δ*C*_1_ = 300 mol m^−3^.

Graphs 2A and 3A as well as 2B and 3B shown in Figure 8 show that in the case of Δ*C*_2_ < 0 and Δ*C*_2_ > 0 (for Δ*C*_1_ = 200 mol m^−3^ and Δ*C*_2_ = 300 mol m^−3^) in ternary solutions, changes in Δ*C*_2_ cause different changes of $J_{2}^{r}$ fluxes. These graphs show that for Δ*C*_2_ < 0, an initial slight linear and then a non-linear decrease in the values of $J_{2}^{r}$ fluxes is observed. Graphs 2B and 3B show that for Δ*C*_2_ > 0, with an increase in the value of Δ*C*_2_, there is a non-linear decrease in the value of the flux $J_{2}^{r}$. Moreover, comparing the graphs 1A, 2A and 3A for the test results presented in this figure, the relations between $J_{2}^{r}$ (for Δ*C*_2_ = 0), $J_{2}^{r}$ (for Δ*C*_1_ = 200 mol m^−3^) and $J_{2}^{r}$ (for Δ*C*_1_ = 300 mol m^−3^) are different depending on the Δ*C*_2_ range. On the other hand, the comparison of graphs 1B, 2B and 3B shows that $J_{2}^{r}$ (for Δ*C*_1_ = 0) < $J_{2}^{r}$ (for Δ*C*_1_ = 200 mol m^−3^) < $J_{2}^{r}$ (for Δ*C*_2_ = 300 mol m^−3^). As in the previous case, the abrupt increase or decrease in the value of $J_{2}^{r}$ is caused by the transition of the system from non-convective to convective state or the other way.

Comparing the curves 2A and 2B as well as 3A and 3B presented in Figure 8, it can be seen that in the case of the first pair of curves, for Δ*C*_2_ = ±476.8 mol m^−3^, the $J_{2}^{r}$ fluxes are equal in value ($J_{2}^{r}$ = ±25.1 × 10^−5^ mol m^−2^s^−1^), but in the opposite direction. In turn, in the case of the second pair of curves, the fluxes $J_{2}^{r}$ are equal in value ($J_{2}^{r}$ = ±35.2 × 10^−5^ mol m^−2^s^−1^) and directed opposite for Δ*C*_2_ = ±664 mol m^−3^. The equality of the volume osmotic flux means that the volume osmotic flux is independent of the configuration of the membrane system. Moreover, for Δ*C*_2_ > −476.8 mol m^−3^ and Δ*C*_2_ > −664 mol m^−3^, membrane transport in configuration A of the membrane system and for Δ*C*_2_ < 476.8 mol m^−3^ and Δ*C*_2_ < 664 mol m^−3^ (for configuration B) takes place under the conditions of concentration polarization destruction by gravitational convection and is diffusive-convective in nature. In turn, for Δ*C*_2_ > −476.8 mol m^−3^ and Δ*C*_2_ > −664 mol m^−3^ in the membrane transport (in configuration A) of the membrane system and for Δ*C*_2_ < 476.8 mol m^−3^ and Δ*C*_2_ < 664 mol m^−3^ (in B of the membrane system) takes place in the conditions of concentration polarization and is diffusive.

**Figure 9:**
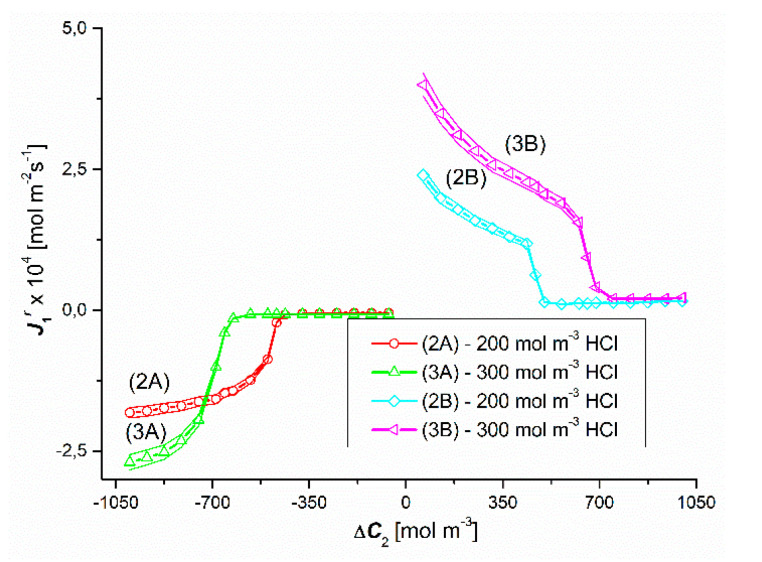
Graphical illustration of the experimental dependence $J_{1}^{r}=f(\Delta C_{2},\;\Delta C_{1}$ = constant), for HCl solutions in NH_4_OH aqueous solution, A and B configurations of the membrane system and concentration polarization conditions. Graphs 2A and 2B—for Δ*C*_1_ = 200 mol m^−3^ and graphs 3A and 3B—for Δ*C*_1_ = 300 mol m^−3^.

Figure 9 shows the results of the $J_{1}^{r}$ flux generated by the constant difference in concentrations $\Delta C_{1}$ = 200 mol m^−3^ (graphs 2A and 2B) and $\Delta C_{1}$ = 300 mol m^−3^ (graphs 3A and 3B ) for a variable value of $\Delta C_{2}$. Hence, $J_{1}^{r}$ should be constant. However, adding ammonia to aqueous HCl solutions reduces the density of the solution, which in turn causes convective movements causing partial destruction of CBLs and leads to an increase in the value of $J_{2}^{r}$, depending on $\Delta C_{1}$.

**Figure 10:**
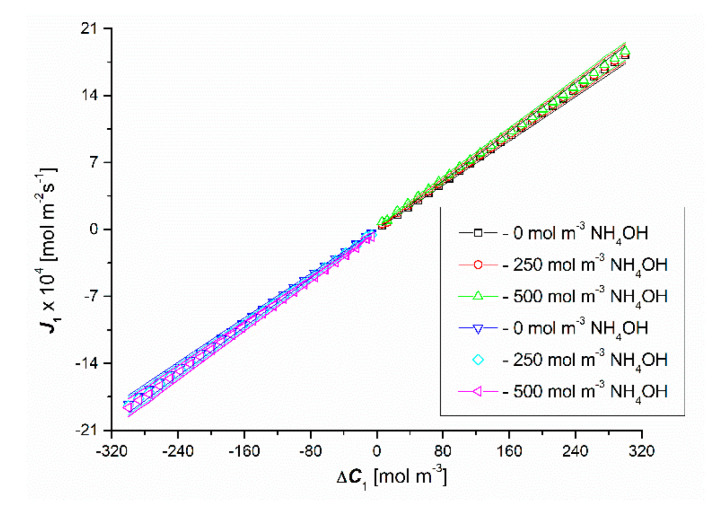
Graphical illustration of the experimental dependence $J_{1}=f(\Delta C_{1},\;\Delta C_{2}$ = constant), for HCl solutions in NH_4_OH aqueous solution of the homogeneity conditions of the solutions.

**Figure 11:**
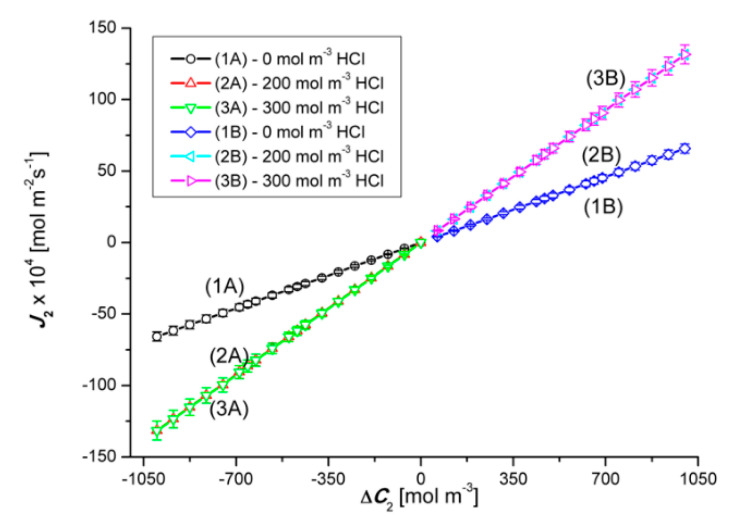
Graphical illustration of the experimental dependence $J_{2}=f(\Delta C_{2},\;\Delta C_{1}$ = constant), for HCl solutions in NH_4_OH aqueous solution and the uniformity conditions of the solutions. Graph 1 was obtained for Δ*C*_1_ = 0, graphs 2A and 2B—for Δ*C*_1_ = 200 mol m^−3^ and graphs 3A and 3B—for Δ*C*_1_ = 300 mol m^−3^.

Figure 10 and Figure 11 show the results of the solute flux tests for the uniformity conditions of the solutions. These figures show that changing the sign of Δ*C*_1_ and/or Δ*C*_2_ changes the sign of $J_{1}$ and $J_{2}$ but does not change the value. This means that $J_{1}$ and $J_{2}$ do not depend on the configuration of the diaphragm system. Moreover, $J_{1}$ is a linear function of Δ*C*_1_, almost independent of the value of Δ*C*_2_.

It should be noted that the addition of 200 mol m^−3^ HCl to aqueous ammonia solutions increases the value of $J_{2}$ by a factor of 2, which is caused by complete dissociation of NH_3_·H_2_O (NH_4_OH) in the presence of HCl. Increasing the HCl concentration to 300 mol m^−3^ does not cause a significant increase in the value of $J_{2}$. For the investigated fluxes, the following relations are satisfied: $J_{v1}$ > $J_{v1}^{r}$ and $J_{1}$ > $J_{1}^{r}$, $J_{v2}$ > $J_{v2}^{r}$ and $J_{2}$ > $J_{2}^{r}$.

For isothermal conditions, due to the density category, binary solutions consisting of water and one dissolved substance can be classified into one of two categories. The first category includes solutions whose density is inversely proportional to their concentration. Examples belonging to this group are aqueous solutions of first order alcohols (methanol, ethanol etc.) and ammonia. The second category is solutions whose density is proportional to their concentration. This category comprises solution not belonging to the first category. Unlike binary solutions, the density of ternary solutions, composed of a solvent and substances causing an increased and decreased solution density (i.e., glucose and ethanol, CuSO_4_ and ethanol, KCl and ammonia or HCl and ammonia, etc.) may be lower than, equal to or greater than that of the solvent [4,8,31].

These trends are evidenced by the same shape of the concentration characteristics of the fluxes for the conditions of concentration polarization. The research shows that the volume fluxes and fluxes of dissolved substances depend on the concentration and composition of solutions (binary or ternary) and the configuration of the membrane system. The presence of alcohol (ethanol, methanol) or ammonia in a ternary solution determines the specificity of the appropriate characteristics for ternary solutions in relation to the appropriate characteristics for binary solutions. It seems that the characteristics for solutions containing HCl and ammonia should be unusual. Because the chemical reaction of HCl + NH_3_∙H_2_O = NH_4_Cl + H_2_O and H^+^ + Cl^−^ + OH^−^ = NH_4_^+^ + Cl^−^ + H_2_O. That is, the product is ammonium chloride. Due to the fact that the density of the aqueous ammonium chloride solution is directly proportional to the concentration, the characteristic should be linear. Research shows otherwise. The concentration characteristics of the streams in the system containing aqueous HCl and NH_3_∙H_2_O solutions are of the same type as the concentration characteristics of the fluxes in the system containing aqueous solutions of glucose and ethanol, KCl and ammonia or CuSO_4_ and ethanol.

### 4.3. Concentration Dependencies of the Global Source of Entropy $\Phi_{Sk}^{r}$ and $\Phi_{Sk}$

**Figure 12:**
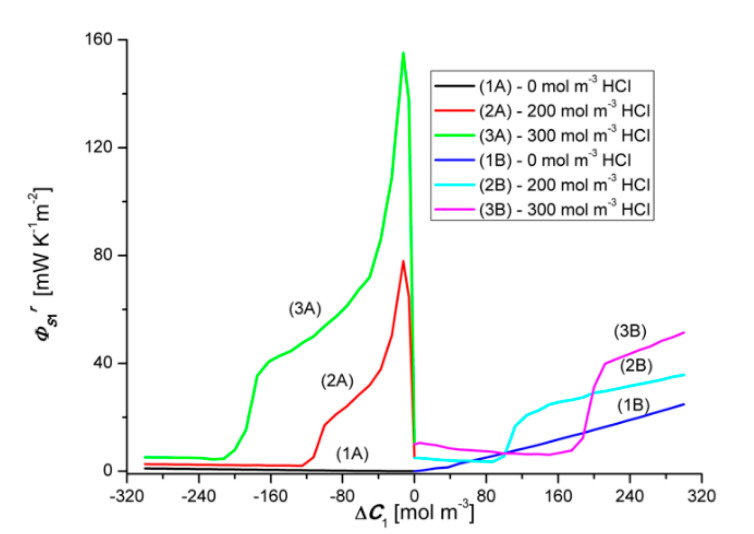
Graphic illustration of the dependence $\Phi_{S1}^{r}=f(\Delta C_{1},\;\Delta C_{2}$ = constant), for HCl solutions in NH_4_OH aqueous solution and concentration polarization conditions. Graphs 1A and 1B were obtained for Δ*C*_2_ = 0, graphs 2A and 2B—for Δ*C*_2_ = 250 mol m^−3^ and graphs 3A and 3B—for Δ*C*_2_ = 500 mol m^−3^.

**Figure 13:**
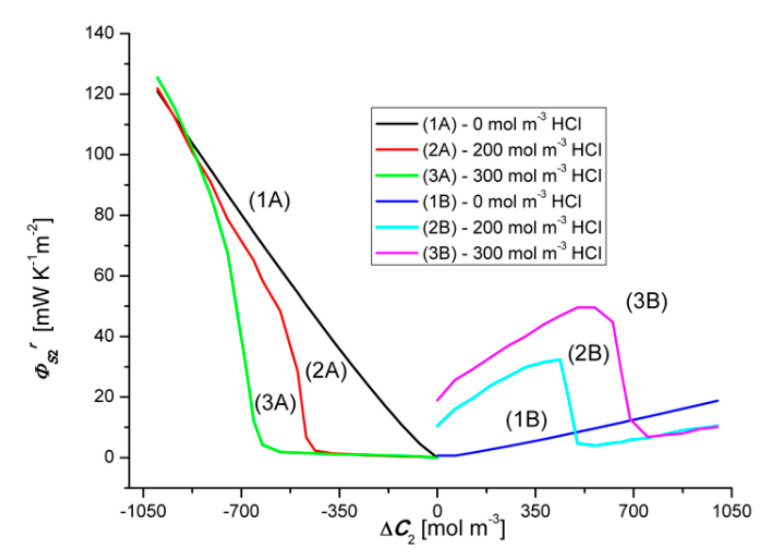
Graphic illustration of the dependence $\Phi_{S2}^{r}=f(\Delta C_{2},\;\Delta C_{1}$ = constant), for NH_4_OH solutions in aqueous HCl solution and concentration polarization conditions. Graphs 1A and 1B were obtained for Δ*C*_1_ = 0, graphs 2A and 2B—for Δ*C*_1_ = 200 mol m^−3^ and graphs 3A and 3B—for Δ*C*_1_ = 300 mol m^−3^.

Equations (1) and (5) show that the global source of entropy $\Phi_{Sk}^{r}$ is the sum of the three components ${\left(\Phi_{Sk}^{r}\right)}_{J_{vk}^{r}}$, ${\left(\Phi_{Sk}^{r}\right)}_{J_{1}^{r}}$ and ${\left(\Phi_{Sk}^{r}\right)}_{J_{2}^{r}}$, while the global source of entropy $\Phi_{Sk}$ is the sum of ${\left(\Phi_{Sk}\right)}_{J_{v1}}$, ${\left(\Phi_{Sk}\right)}_{J_{1}}$ and ${\left(\Phi_{Sk}\right)}_{J_{2}}$ (*k* = 1, 2). Figure 12 and Figure 13 show the dependencies $\Phi_{S1}^{r}=f(\Delta C_{1},\;\Delta C_{2}$ = constant) and $\Phi_{S2}^{r}=f(\Delta C_{2},\;\Delta C_{1}$ = constant), calculated on the basis of Equation (9) and the experimental $J_{v1}^{r}=f(\Delta C_{1},\;\Delta C_{2}$ = constant), $J_{v2}^{r}=f(\Delta C_{2},\;\Delta C_{1}$ = constant), $J_{1}^{r}=f(\Delta C_{1},\;\Delta C_{2}$ = constant) and $J_{2}^{r}=f(\Delta C_{2},\;\Delta C_{1}$ = constant).

Graph 1B presented in Figure 12 shows that $\Phi_{S1}^{r}$ increases linearly with the increase of the value of $\Delta C_{1}$. On the other hand, graph 1A shows that changing the sign of $\Delta C_{1}$ from positive to negative also causes a linear increase of $\Phi_{S1}^{r}$ but its values, in the case of negative $\Delta C_{1}$, are much smaller compared to the value of $\Phi_{S1}^{r}$ for positive $\Delta C_{1}$. The dependence $\Phi_{S1}^{r}=f(\Delta C_{1},\;\Delta C_{2}$ = constant), Illustrated by the curves 2B and 3B, have a similar shape and are nonlinear. Two areas can be separated in the course of these curves. The first, where $\Phi_{S1}^{r}$ is weakly dependent on $\Delta C_{1}$, related to the osmotic-diffusion production of entropy, and the second, where $\Phi_{S1}^{r}$ is strongly dependent on $\Delta C_{1}$, related to the osmotic-diffusion-convective production of entropy.

The dependences $\Phi_{S1}^{r}=f(\Delta C_{1},\;\Delta C_{2}$ = constant), illustrated by the curves 2A and 3A, have a similar shape and are nonlinear. Two areas can also be separated in the course of these curves. The first, where $\Phi_{S1}^{r}$ is strongly dependent on $\Delta C_{1}$, related to the osmotic-diffusion-convective production of entropy, and the second, where $\Phi_{S1}^{r}$ is weakly dependent on $\Delta C_{1}$, related to the osmotic-diffusion production of entropy. Moreover, it can be seen from Figure 12 and Figure 13 that the Charts 1A, 2A, 3A are asymmetric with respect to the Charts 1B, 2B and 3B with respect to the vertical axis passing through the zero point.

Graph 1B presented in Figure 13 shows that $\Phi_{S2}^{r}$ increases linearly with the increase of the value of $\Delta C_{2}$. Graph 1A, in turn, shows that the change of the sign of $\Delta C_{1}$ from positive to negative also causes a linear increase of $\Phi_{S2}^{r}$, but its values, in the case of negative $\Delta C_{2}$, are much larger compared to the value of $\Phi_{S2}^{r}$ for positive $\Delta C_{2}$. The dependences $\Phi_{S2}^{r}=f(\Delta C_{2},\;\Delta C_{1}$ = constant), Illustrated by the curves 2B and 3B, have a similar shape and are nonlinear. Two areas can be separated in the course of these curves. The first, where $\Phi_{S2}^{r}$ are strongly dependent on $\Delta C_{2}$, related to the osmotic-diffusion-convective production of entropy, and the second, where $\Phi_{S2}^{r}$ is weakly dependent on $\Delta C_{2}$, related to the osmotic-diffusion production of entropy. The dependences $\Phi_{S2}^{r}=f(\Delta C_{2},\;\Delta C_{1}$ = constant), illustrated by the curves 2A and 3A, have a similar shape and are non-linear. Two areas can also be separated in the course of these curves. The first, where $\Phi_{S2}^{r}$ is weakly dependent on $\Delta C_{2}$, related to the osmotic-diffusion production of entropy, and the second, where $\Phi_{S2}^{r}$ is strongly dependent on $\Delta C_{2}$, related to the osmotic-diffusion-convective production of entropy.

**Figure 14:**
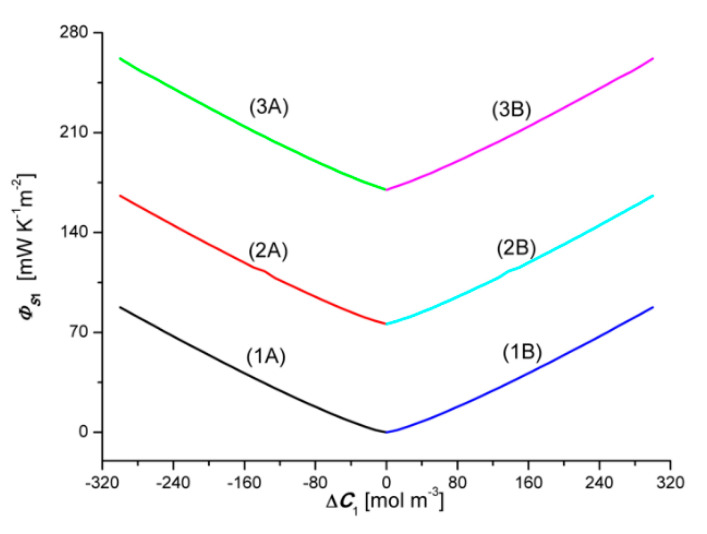
Graphic illustration of the dependence $\Phi_{S1}=f(\Delta C_{2},\;\Delta C_{1}$ = constant), for HCl solutions in NH_4_OH aqueous solution and the uniformity conditions of the solutions. Graphs 1A and 1B were obtained for Δ*C*_1_ = 0, graphs 2A and 2B—for Δ*C*_1_ = 200 mol m^−3^ and graphs 3A and 3B—for Δ*C*_1_ = 300 mol m^−3^.

**Figure 15:**
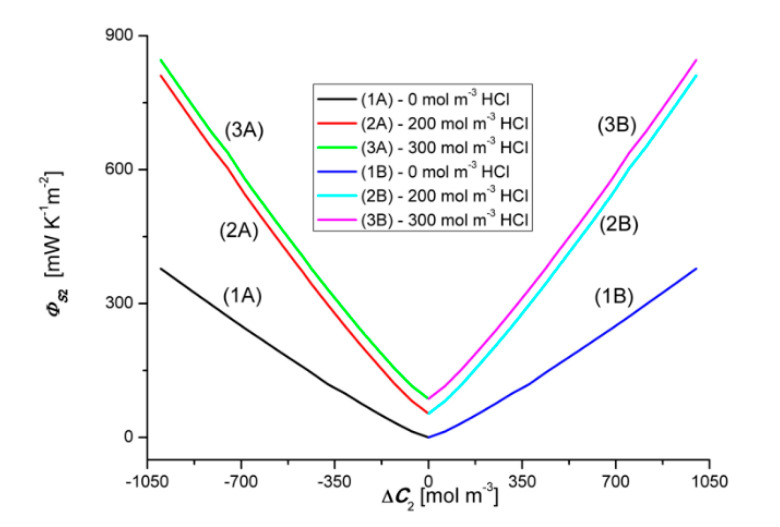
Graphic illustration of the dependence $\Phi_{S2}=f(\Delta C_{2},\;\Delta C_{1}$ = constant), for NH_4_OH solutions in an aqueous HCl solution and homogeneity conditions of the solutions. Graphs 1A and 1B were obtained for Δ*C*_1_ = 0, graphs 2A and 2B—for Δ*C*_1_ = 200 mol m^−3^ and graphs 3A and 3B—for Δ*C*_1_ = 300 mol m^−3^.

Figure 14 and Figure 15 show the dependencies $\Phi_{S1}=f(\Delta C_{1},\;\Delta C_{2}$ = constant) and $\Phi_{S2}=f(\Delta C_{2},\;\Delta C_{1}$ = constant), respectively, for the conditions homogeneity of solutions, calculated on the basis of Equation (10) and the experimental dependencies $J_{v1}=f(\Delta C_{1},\;\Delta C_{2}$ = constant), $J_{v2}=f(\Delta C_{2},\;\Delta C_{1}$ = constant), $J_{1}=f(\Delta C_{1},\;\Delta C_{2}$ = constant) i $J_{2}=f(\Delta C_{2},\;\Delta C_{1}$ = constant). Graphs 1B, 2B and 3B shown in Figure 14 show that $\Phi_{S1}$ increases with the increase of the value of $\Delta C_{1}$. Changing the sign of $\Delta C_{1}$ does not change the value of $\Phi_{S1}$. Adding a constant amount of NH_4_OH to aqueous HCl solutions causes a shift of plots 2A and 2B with respect to plots 1A and 1B and plots 3A and 3B against plots 2A and 2B.

Graphs 1B, 2B and 3B shown in Figure 15 show that $\Phi_{S2}$ increases with the increase of the value of $\Delta C_{2}$. Changing the sign of $\Delta C_{2}$ does not change the value of $\Phi_{S2}$. Adding a constant amount of HCl to aqueous NH_4_OH solutions causes a shift of plots 2A and 2B with respect to plots 1A and 1B, and plots 3A and 3B against plots 2A and 2B. The comparison of the graphs presented in Figure 12 and Figure 13 shows that the graphs 1A, 2A, and 3A are symmetrical to the graphs 1B, 2B and 3B about the vertical axis passing through the zero point. There are relations between the above-mentioned quantities $\Phi_{S1}$ > $\Phi_{S1}^{r}$, $\Phi_{S2}$ >$\Phi_{S2}^{r},$ ${\left(\Phi_{S1}\right)}_{J_{v1}}\;>\;{\left(\Phi_{S1}\right)}_{J_{v1}^{r}}$, ${\left(\Phi_{S2}\right)}_{J_{v2}}\;{\left(\Phi_{S2}\right)}_{J_{v2}^{r}}$ ${\left(\Phi_{S1}\right)}_{J_{1}}$ > ${\left(\Phi_{S1}^{r}\right)}_{J_{1}^{r}}$ and ${\left(\Phi_{S2}\right)}_{J_{2}}$ > ${\left(\Phi_{S2}^{r}\right)}_{J_{2}^{r}}$. The largest share in $\Phi_{Sk}^{r}$ are the components ${\left(\Phi_{Sk}^{r}\right)}_{J_{1}^{r}}$ and ${\left(\Phi_{Sk}^{r}\right)}_{J_{2}^{r}}$ and in the case of $\Phi_{Sk}—{\left(\Phi_{Sk}\right)}_{J_{1}}$ and ${\left(\Phi_{Sk}\right)}_{J_{2}}$ (*k* = 1, 2).

### 4.4. Concentration Dependences Diffusion-Convective Effect $\Delta \Phi_{Sk}^{r}$

To calculate the difference ($\Delta \Phi_{Sk}^{r}$) between the entropy source for the uniformity conditions of the solutions ($\Phi_{Sk}$) and the concentration polarization conditions ($\Phi_{Sk}^{r}$) we use the following equation:

(11) $$\Phi_{Sk}^{r}=\Phi_{Sk}-\Phi_{Sk}^{r}$$

and the dependencies $\Phi_{S1}=f(\Delta C_{2},\;\Delta C_{1}$ = constant), $\Phi_{S2}=f(\Delta C_{2},\;\Delta C_{1}$ = constant), $\Phi_{S1}^{r}=f(\Delta C_{1},\;\Delta C_{2}$ = constant), The dependencies $\Phi_{S2}^{r}=f(\Delta C_{2},\;\Delta C_{1}$ = constant), shown in Figure 12, Figure 13, Figure 14 and Figure 15. The difference $\Delta \Phi_{Sk}^{r}$ is a measure of the diffusion-convective effect.

**Figure 16:**
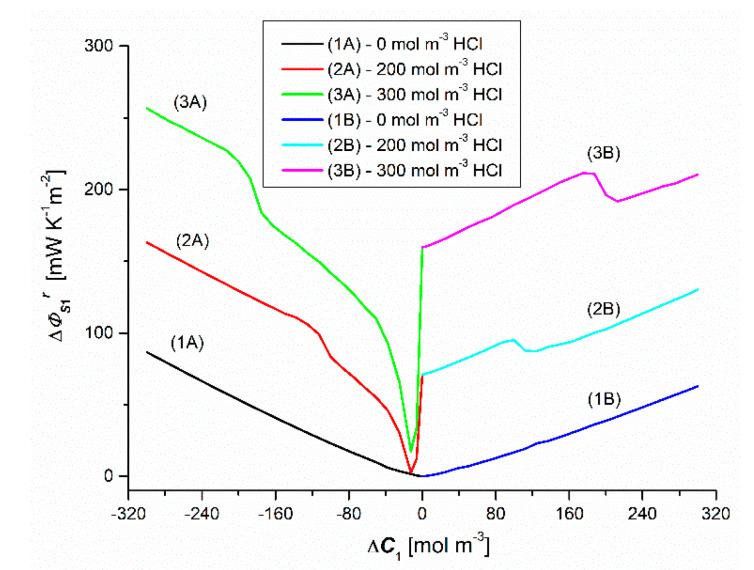
Graphic illustration of the relationship $\Delta \Phi_{S1}^{r}=f(\Delta C_{1},\;\Delta C_{2}$ = constant), for HCl solutions in NH_4_OH aqueous solution and concentration polarization conditions. Graphs 1A and 1B were obtained for Δ*C*_2_ = 0, graphs 2A and 2B—for Δ*C*_2_ = 250 mol m^−3^ and graphs 3A and 3B—for Δ*C*_2_ = 500 mol m^−3^.

Figure 16 shows the dependencies $\Delta \Phi_{S1}^{r}=f(\pm \Delta C_{1},\;\pm \Delta C_{2}$ = const.), calculated on the basis of Equation (11), taking into account the dependencies $\Phi_{S1}=f(\Delta C_{1},\;\Delta C_{2}$ = const.) and $\Phi_{S1}^{r}=f(\Delta C_{1},\;\Delta C_{2}$ = const.), presented in Figure 10 and Figure 12. The figures shows that the curves 1A, 2A and 3A are asymmetric with respect to the curves 1B, 2B and 3B with respect to the vertical axis crossing zero.

**Figure 17:**
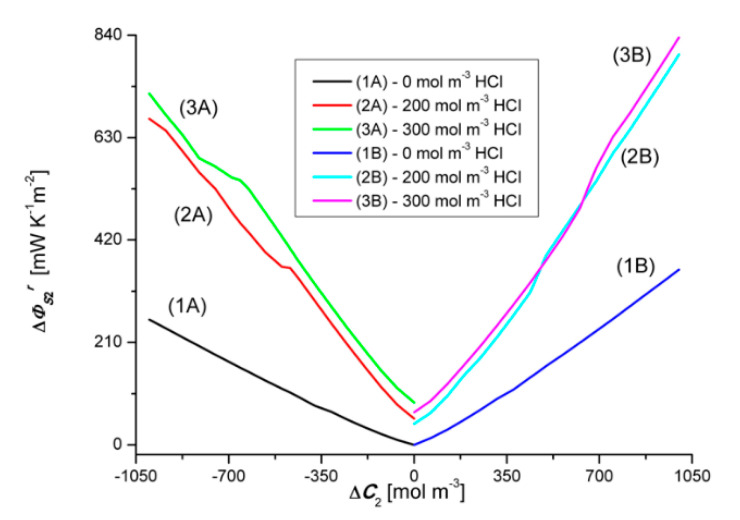
Graphic illustration of the dependence $\Delta \Phi_{S2}^{r}=f(\Delta C_{2},\;\Delta C_{1}$ = constant), (*r* = A, B) for NH_4_OH solutions in aqueous HCl solution and concentration polarization conditions. Graphs 1A and 1B were obtained for Δ*C*_1_ = 0, graphs 2A and 2B—for Δ*C*_1_ = 200 mol m^−3^ and graphs 3A and 3B—for Δ*C*_1_ = 300 mol m^−3^.

Figure 17 shows the dependencies $\Delta \Phi_{S2}^{r}=f(\Delta C_{2},\;\Delta C_{1}$ = constant), calculated on the basis of Equation (12), taking into account the dependencies $\Phi_{S2}=f(\Delta C_{2},\;\Delta C_{1}$ = constant) and $\Phi_{S2}^{r}=f(\Delta C_{2},\;\Delta C_{1}$ = constant), presented in Figure 11 and Figure 13. This figure shows that the curves 1A, 2A and 3A are asymmetric with respect to the curves 1B, 2B and 3B with respect to the vertical axis passing through zero.

### 4.5. Concentration Dependencies of the Convective Polarization Effect

To calculate the convective effects $\alpha_{k}$ we use Equation (12):

(12) $$\alpha_{k}=\Phi_{Sk}^{A}-\Phi_{Sk}^{B}\;(k\;=\;1,\;2)$$

**Figure 18:**
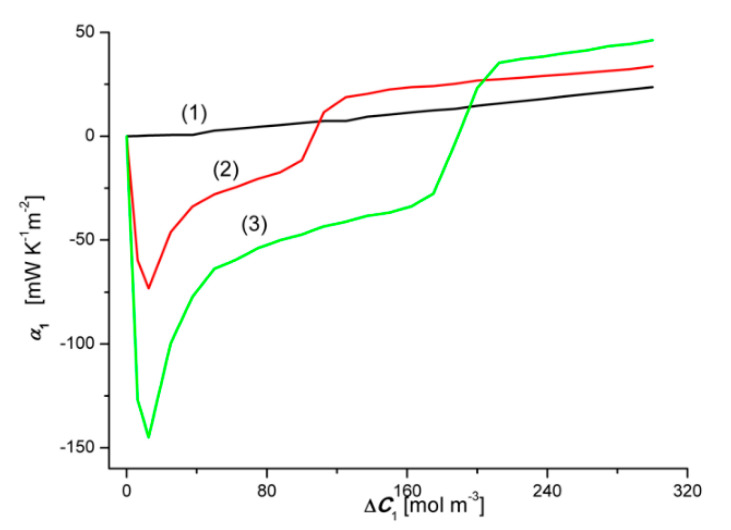
Graphical illustration of the relationship $\alpha_{1}=f(\Delta C_{1},\;\Delta C_{2}$ = constant), for HCl solutions in NH_4_OH aqueous solution and concentration polarization conditions. Graphs 1A and 1B were obtained for Δ*C*_2_ = 0, graphs 2A and 2B—for Δ*C*_2_ = 250 mol m^−3^ and graphs 3A and 3B—for Δ*C*_2_ = 500 mol m^−3^.

**Figure 19:**
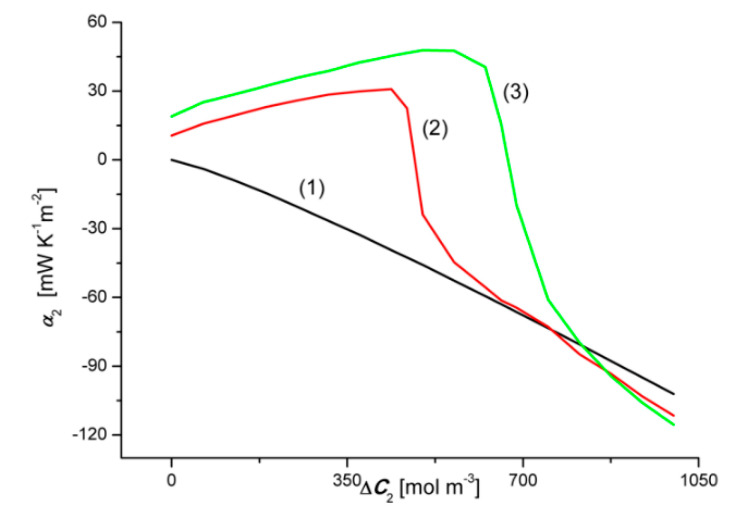
Graphical illustration of the relationship $\alpha_{2}=f(\Delta C_{2},\;\Delta C_{1}$ = constant), for NH_4_OH solutions in aqueous HCl solution and concentration polarization conditions. Graphs 1A and 1B were obtained for Δ*C*_1_ = 0, graphs 2A and 2B—for Δ*C*_1_ = 200 mol m^−3^ and graphs 3A and 3B—for Δ*C*_1_ = 300 mol m^−3^.

Concentration dependencies of the source of entropy $\alpha_{1}=f(\Delta C_{1},\;\Delta C_{2}$ = constant) and $\alpha_{2}=f(\Delta C_{2},\;\Delta C_{1}$ = constant), illustrated by graphs 1A, 2A, 3A and 1B, 2B and 3B are shown in Figure 10 and Figure 11. The calculation results are presented in Figure 18 and Figure 19.

Figure 18 and Figure 19 show that the relationships $\alpha_{1}=f(\Delta C_{1},\;\Delta C_{2}$ = 0) and $\alpha_{2}=f(\Delta C_{2},\;\Delta C_{1}$ = 0), are linear, as illustrated by graphs 1, where $\alpha_{1}$ > 0, while $\alpha_{2}$ < 0 and $\alpha_{1}$ < $\left|\alpha_{2}\right|$ in the whole range of tested solution concentration differences. Negative $\alpha_{1}$ means that the convection currents are directed vertically downwards. In turn, positive $\alpha_{2}$ informs that convection currents are directed vertically upwards. In the case of the dependences $\alpha_{1}=f(\Delta C_{1},\;\Delta C_{2}$ > 0) and $\alpha_{2}=f(\Delta C_{2},\;\Delta C_{1}$ > 0), both $\alpha_{1}$ and $\alpha_{2}$ can be negative, positive or equal to zero. This means that with a change in the sign of $\alpha_{1}$ or $\alpha_{2}$, the sense of convection currents changes: in the case of $\alpha_{1}$, from vertical up to vertical down, and in the case of $\alpha_{2}$—from vertical down to vertical up. Similar results as in Figure 18 and Figure 19 were obtained for aqueous CuSO_4_ and/or ethanol solutions [4].

### 4.6. Evaluation of the Coefficients $\zeta_{k}^{r}$ and Katchalsky Number (Ka)

**Figure 20:**
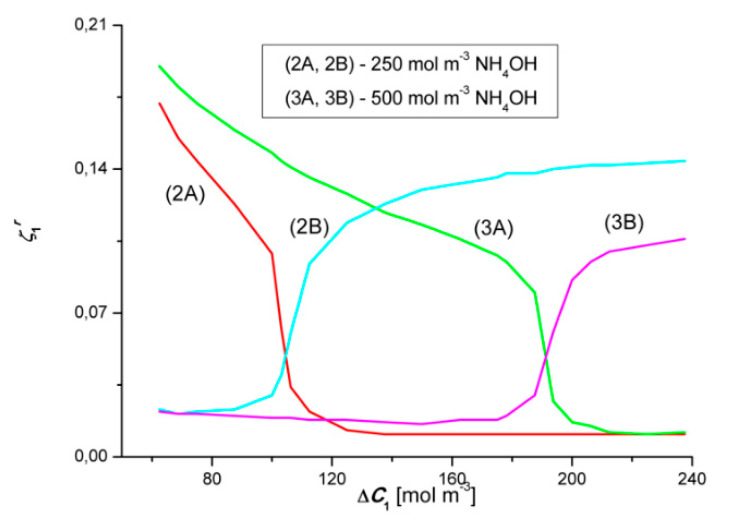
Graphical illustration of the dependence $\zeta_{1}^{r}=f(\Delta C_{1},\;\Delta C_{2}$ = constant) (*r* = A, B) for HCl solutions in NH_4_OH aqueous solution and concentration polarization conditions. Graphs 2A and 2B—for Δ*C*_2_ = 250 mol m^−3^ and graphs 3A and 3B—for Δ*C*_2_ = 500 mol m^−3^.

**Figure 21:**
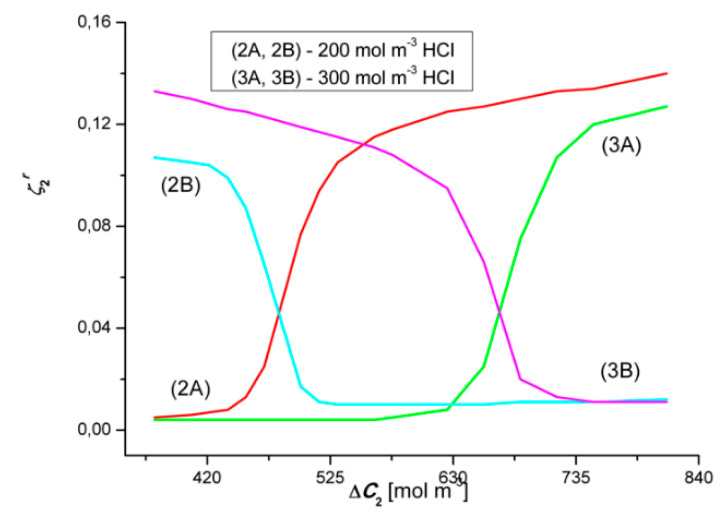
Graphical illustration of the dependence $\zeta_{2}^{r}=f(\Delta C_{2},\;\Delta C_{1}$ = constant), (*r* = A, B) for NH_4_OH solutions in an aqueous HCl solution and concentration polarization conditions. Graphs 2A and 2B—for Δ*C*_1_ = 200 mol m^−3^ and graphs 3A and 3B—for Δ*C*_1_ = 300 mol m^−3^.

Figure 20 and Figure 21 show the concentration dependencies of the concentration polarization coefficients of the dependence $\zeta_{1}^{r}$ and $\zeta_{2}^{r}$. These coefficients are defined by the expressions: $\zeta_{1}^{r}$ = $J_{v1}^{r}/J_{v1}$ = $J_{1}^{r}/J_{1}$ and $\zeta_{2}^{r}$ = $J_{v2}^{r}/J_{v2}$ = $J_{2}^{r}/J_{2}$. The dependencies $\zeta_{1}^{r}=f(\Delta C_{1},\;\Delta C_{2}$ = constant) and $\zeta_{2}^{r}=f(\Delta C_{2},\;\Delta C_{1}$ = constant), calculated on the basis of the test results shown in Figure 2, Figure 3, Figure 4, Figure 5, Figure 6, Figure 7, Figure 8, Figure 9, Figure 10 and Figure 11. Figure 20 shows that the relationships 2A and 2B intersect at the coordinates $\zeta_{1}$ = 0.042 and Δ*C*_1_ = 105.85 mol m^−3^, while the relationships 3A and 3B—at the point with the coordinates $\zeta_{1}$ = 0.045 and Δ*C*_1_ ≈ 190 mol m^−3^. These points correspond to the points where the convective effect disappears, as measured by the coefficient $\alpha_{1}$. Figure 18 shows that $\alpha_{1}$ = 0 for Δ*C*_1_ ≈ 106 mol m^−3^ and Δ*C*_1_ ≈ 188 mol m^−3^. As already mentioned, the convective effect appears for $\alpha_{1}$ < 0 and $\alpha_{1}$ > 0.

Figure 21 shows that the graphs 2A and 2B intersect at the coordinates $\zeta_{2}$ ≈ 0.045 and Δ*C*_2_ ≈ 480 mol m^−3^, while the relationships 3A and 3B−at the coordinates $\zeta_{2}$ ≈ 0.045 and Δ*C*_2_ ≈ 670 mol m^−3^. These points correspond to the points where the convective effect disappears, as measured by the coefficient $\alpha_{2}$. Figure 19 shows that $\alpha_{2}$ = 0 for Δ*C*_2_ ≈ 483 mol m^−3^ and Δ*C*_2_ ≈ 672 mol m^−3^. In this case, the convective effect appears for $\alpha_{2}$ > 0 and $\alpha_{2}$ < 0. This means that the points where $\alpha_{1}$ = 0 and $\alpha_{2}$ = 0 are compatible with the critical value of the coefficient $\zeta_{1}$ and/or $\zeta_{2}$. Typically, the Rayleigh concentration number ($R_{Ck}^{r}$) is used as the control parameter. We propose to call this expression the Katchalsky number (*Ka*). Let us consider Equation (7) and transform it to the form:

(13) $$\frac{\rho_{0}}{\sum_{k=1}^{2}\left(\frac{\partial \rho }{\partial C_{k}}\right)\left(C_{hk}-C_{lk}\right)}R_{Ck}=\frac{gD_{k}{}^{2}}{16\nu_{0}{\left(RT\omega_{k}\right)}^{3}}\frac{{\left(1-\zeta_{k}\right)}^{4}}{\zeta_{k}{}^{3}}\equiv \;Ka$$

We denote the left side of the equation by *Ka* and we propose to call it the Katchalsky Number. Taking into account the table data: *g* = 9.81 m s^−2^, *R* = 8.31 J mol^−1^K^−1^, *T* = 295 K, *D*_1_ = 2.43 × 10^−9^ m^2^s^−1^, *D*_2_ = 3.78 × 10^−9^ m^2^s^−1^, *ω*_1_ = 1.24 × 10^−9^ m^2^s^−1^, *ω*_2_ = 1.68 × 10^−9^ m^2^s^−1^, *ω*_0_ = 1.012 × 10^−6^ m^2^s^−1^ $\zeta_{1}$ = $\zeta_{2}$ = 0.045, we get *KS*_1_ = 1.16 × 10^9^, *KS*_2_ = 1.13 × 10^9^.

The $\zeta_{k}^{r}$ coefficient may take values in the range 0 ≤ $\zeta_{k}^{r}$ ≤ 1. For 0 ≤ $\zeta_{k}^{r}$ ≤ $\zeta_{k}$ we are dealing with a gravitational convection. If $\zeta_{k}^{r}$ takes values in the range $\zeta_{k}$ < $\zeta_{k}^{r}$ ≤ ${\left(\zeta_{k}^{r}\right)}_{max}$, the state of gravitational convection occurs in the membrane system. If, on the other hand, $\zeta_{k}^{r}$ takes values in the range ${\left(\zeta_{k}^{r}\right)}_{max}$ < $\zeta_{k}^{r}$ ≤ 1, the system is in the state of forced convection. This means that the greater the value of $\zeta_{k}^{r}$, the smaller the value of *Ka*.

## 5. Conclusions

In the paper, the authors present the results of research on the effects of the concentration and orientation of aqueous HCl and/or ammonia solutions in relation to a horizontally oriented membrane, under Earth gravity conditions, on the value of osmotic volume fluxes ($J_{vk}^{r})$ and dissolved substances $(J_{k}^{r}$). It has been shown that for the polarization conditions of the concentration and of aqueous HCl or ammonia solutions, $J_{vk}^{r}$ and $J_{k}^{r}$ are linear, and for aqueous HCl and ammonia solutions, non-linear functions of solution concentration differences. Moreover, it has been shown that the values of $J_{vk}^{r}$ and $J_{k}^{r}$ depend on the alignment of the solutions with respect to the horizontally oriented membrane. In the case of mechanically stirred solutions, $J_{vk}$ and $J_{k}$ are independent of the orientation of the solutions in relation to the horizontally oriented membrane and are a linear function of the difference in concentrations of the solutions of both aqueous HCl or ammonia solutions and aqueous HCl and ammonia solutions. For the investigated fluxes, the following relations are satisfied: $J_{vk}$ > $J_{vk}^{r}$ and $J_{k}$ > $J_{k}^{r}$.

A common feature of the $J_{vk}^{r}$ and $J_{k}^{r}$ concentration relationships for aqueous HCl and/or ammonia solutions is the change in the nature of transport from osmotic-diffusion to osmotic-diffusion-convective or the other way around. This means that under the Earth’s gravitational field conditions and concentration field dependency on the density of the solutions separated by the membrane, gravitational convection appears or disappears. The measure of the effect of gravitational convection is the coefficient $\alpha_{k}$, which can take positive or negative values. A positive value of this coefficient indicates that the convective movements that destroy CBLs are vertically downward, and negative—vertically upward. The transition from non-convective to convective or the other way has the characteristics of a pseudo-phase transition. All the above-mentioned features have a global source of entropy ($\Phi_{Sk}^{r}$), which for solutions containing a solvent and two dissolved substances is the sum of three partial sources of entropy, the global source of entropy is the sum of three components ${\left(\Phi_{Sk}^{r}\right)}_{J_{vk}^{r}}$, ${\left(\Phi_{Sk}^{r}\right)}_{J_{1}^{r}}$ and ${\left(\Phi_{Sk}^{r}\right)}_{J_{2}^{r}}$, (*k* = 1, 2). It is similar in the case of homogeneous solutions: the global source of entropy $\Phi_{Sk}$ is the sum of ${\left(\Phi_{Sk}\right)}_{J_{v1}}$, ${\left(\Phi_{Sk}\right)}_{J_{1}}$ and ${\left(\Phi_{Sk}\right)}_{J_{2}}$ (*k* = 1, 2). There are relations between the above-mentioned quantities $\Phi_{Sk}$ > $\Phi_{Sk}^{r}$, ${\left(\Phi_{Sk}\right)}_{J_{v1}}$ > ${\left(\Phi_{Sk}\right)}_{J_{v1}}$, ${\left(\Phi_{Sk}\right)}_{J_{1}}$ > ${\left(\Phi_{Sk}^{r}\right)}_{J_{1}^{r}}$ and ${\left(\Phi_{Sk}\right)}_{J_{2}}$ > ${\left(\Phi_{Sk}^{r}\right)}_{J_{2}^{r}}$, (*k* = 1, 2). The largest share in $\Phi_{Sk}^{r}$ are the components ${\left(\Phi_{Sk}^{r}\right)}_{J_{1}^{r}}$ and ${\left(\Phi_{Sk}^{r}\right)}_{J_{2}^{r}}$ and in the case of $\Phi_{Sk}—{\left(\Phi_{Sk}\right)}_{J_{1}}$ and ${\left(\Phi_{Sk}\right)}_{J_{2}}$.

It has been shown that the coefficient $\zeta_{i}^{r}$ can be related to the concentration number Rayleigh $\left(R_{Ck}\right)$, i.e., with the parameter controlling the transition from the non-convective (diffusive) state to the convective state. The article uses an innovative approach consisting in replacing the expression $R_{Ck}\rho_{0}/\sum_{k=1}^{2}\left(\frac{\partial \rho }{\partial C_{k}}\right)\left(C_{hk}-C_{lk}\right)$ with a Katchalsky number (*Ka*):

$$Ka=\frac{gD_{k}{}^{2}}{16\nu_{0}{\left(RT\omega_{k}\right)}^{3}}\frac{{\left(1-\zeta_{k}\right)}^{4}}{\zeta_{k}{}^{3}}$$

This number acts as a switch between the two states of the concentration field: convective (with a higher entropy source value) and non-convective (with a lower entropy source value). The operation of this switch indicates the regulatory role of Earth’s gravity in relation to membrane transport.

This number acts as a switch between two states of the concentration field: convective (with a higher entropy source value) and convection-less (with a lower entropy source value). The operation of this switch indicates the regulatory role of Earth’s gravity in relation to membrane transport.

## References

[1] Dermirel Y. (2007). Nonequilibrium Thermodynamics: Transport and Rate Processes in Physical, Chemical and Biological Systems.

[2] Cheng X., Pinsky P.M. (2015). The balance of fluid and osmotic pressures across active biological membranes with application to the corneal endothelium. PLoS ONE.

[3] Delmotte M., Chanu J., Millazzo G. (1979). Non-equilibrium thermodynamics and membrane potential measurement in biology. Topics Bioelectrochemistry and Bioenergetics.

[4] Batko K.M., Ślęzak A., Bajdur W.M. (2020). The role of gravity in the evolution of the concentration field in the electrochemical membrane cel. Entropy.

[5] Abu-Rjal R., Prigozchin L., Rubinstein I., Zaltzman B. (2015). Teorell instability in concentration polarization. Phys. Rev. E.

[6] Mishchuk N.A. (2010). Concentration polarization of interface and non-linear electrokinetic phenomena. Adv. Colloid Interface Sci..

[7] Nikonenko V., Nebravsky A., Mareev S., Kovalenko A., Urtenov M., Pourcelly G. (2019). Modelling of ion transport in electromembrane systems: Impact of membrane bulk and surface heterogeneity. Appl. Sci..

[8] Ślęzak A. (1989). Irreversible thermodynamic model equations of the transport across a horizontally mounted membrane. Biophys. Chem..

[9] Ślęzak A. (1990). A Model equation for the gravielectric effect in electrochemical cells. Biophys. Chem..

[10] Lipton B. (2018). The Biology of Belief: Unleashing the Power of Consciousness.

[11] Baker R. (2012). Membrane Technology and Application.

[12] Uragami T. (2017). Science and Technology of Separation Membranes.

[13] Nunes S.P., Culfaz-Emecen P.Z., Ramon G.Z., Visser T., Koops G.H., Jin W., Ulbricht M. (2020). Thinking the future of membranes: Perspectives for advanced and new membrane materials and manufacturing processes. J. Membr. Sci..

[14] Nguyen T.P.N., Jun B.M., Lee J.H., Kwon Y.-M. (2015). Comparison of integrally asymmetric and thin film composite structures for a desirable fashion of forward osmosis membranes. J. Membr. Sci..

[15] Nga Nguyen T.P., Byung-Moon N., Kwon Y.N. (2017). The chlorination mechanism of integrally asymmetric cellulose triacetate (CTA)-based and thin film composite polyamide-based forward osmosis membranes. J. Membr. Sci..

[16] Lakshminarayanaiah N. (1969). Transport Phenomena in Membanes.

[17] Katchalsky A., Curran P.F. (1965). Nonequilibrium Thermodynamics in Biophysics.

[18] Peusner L. (1986). Studies in Network Thermodynamics.

[19] Mason E.A., Viehland L.A. (1978). Statistical-mechanical theory of membranę transport for multicomponent systems: Passive transport through open membranes. J. Chem. Phys..

[20] Mehta G.D., Morse E.A., Mason E.A., Daneshpajooh M.H. (1976). Generalized Nernst-Planck and Stefan-Maxwell equations for membrane transport. J. Chem. Phys..

[21] Hall M.S., Starov V.M., Lloyd D.R. (1997). Reverse osmosis of multicomponent electrolyte solutions. Part I. Theoretical development. J. Membr. Sci..

[22] Hall M.S., Lloyd D.R., Starov V.M. (1997). Reverse osmosis of multicomponent electrolyte solutions. Part II. Experimental verification. J. Membr. Sci..

[23] Batko K.M., Ślęzak-Prochazka I., Grzegorczyn S., Ślęzak A. (2014). Membrane transport in concentration polarization conditions: Network thermodynamics model equations. J. Porous. Media.

[24] Réjou-Michel A., Vilardi M., Delmotte M. (1979). Contributions of the electric potential difference of a membranę system under clamped ionic gradient. J. Electroanal. Chem. Interfac. Electochem..

[25] Ślęzak A., Ślęzak-Prochazka I., Grzegorczyn S., Jasik-Ślęzak J. (2017). Evaluation of S-Entropy production in a single-membrane system in concentration polarization conditions. Trans. Porous Med..

[26] Batko K.M., Ślęzak A. (2019). Membrane transport of nonelectrolyte solutions in concentration polarization condition: H^r^ form of the Kedem-Katchalsky-Peusner equations. Inter. J. Chem. Eng..

[27] Ślęzak A., Grzegorczyn S., Batko M., Pilis W., Biczak R. (2020). Membrane transport in concentration polarization conditions: Evaluation of S-entropy production for ternary non-electrolyte solutions. J. Non-Equilib. Thermodyn..

[28] Dworecki K., Ślęzak A., Ornal-Wąsik B., Wąsik S. (2005). Effect of hydrodynamic instabilities on solute transport in membrane system. J. Membr. Sci..

[29] Ślęzak A., Dworecki K., Ślęzak I.H., Wąsik S. (2005). Permeability coefficient model equations of the complex: Membrane-concentration boundary layers for ternary nonelectrolyte solutions. J. Membr. Sci..

[30] Ślęzak A., Grzegorczyn S., Jasik-Ślęzak J., Michalska-Małecka K. (2010). Natural convection as an asymmetrical factor of the transport through porous membrane. Transp. Porous Media.

[31] Ślęzak A., Dworecki K., Anderson J.E. (1985). Gravitational effects on transmembrane flux: The Rayleigh-Taylor convective instability. J. Membr. Sci..

[32] Dworecki K., Wąsik S., Ślęzak A. (2003). Temporal and spatial structure of the concentration boundary layers in membrane system. Physica A.

[33] Lohaus T., Herkenhoff N., Shankar R., Wessing M. (2018). Feed flow patterns of combined Rayleigh-Bénard convection and membrane permeation. J. Membr. Sci..

[34] Lebon G., Jou D., Casas-Vasquez J. (2008). Understanding Non-Equilibrium Thermodynamics. Foundations, Applications, Frontiers.

[35] Jasik-Ślęzak J., Olszówka K.M., Ślęzak A. (2011). Estimation of thickness of concentration boundary layers by osmotic volume flux determination. Gen. Physiol. Biophys..

[36] Ślęzak A., Dworecki K., Jasik-Ślęzak J., Wąsik J. (2004). Method to determine the practical concentration Rayleigh number in isothermal passive membrane transport processes. Desalination.

[37] Puthenveettil B.A., Arakeri J.H. (2005). Plum structure in high-Rayleigh-Number convection. J. Fluid Mech..

[38] Puthenveettil B.A., Gunasegarane G.S., Agrawal Y.K., Schmeling D., Bosbach J., Arakeri J.H. (2011). Length of near-wall plumes in turbulent Convection. J. Fluid Mech..

[39] Klinkman H., Holtz M., Willgerodt W., Wilke G., Schoenfelder D. (1969). Nephrophan—Eine neue dialysemembran. Z. Urol. Nephrol..

[40] Cammann K. (1973). Das Arbeiten Mit Ionencelektiven Electroden. Eine Einführung.

[41] Ewing G.W. (1985). Instrumental Methods of Chemical Analysis.

